# The pattern of alternative splicing and DNA methylation alteration and their interaction in linseed (*Linum usitatissimum* L.) response to repeated drought stresses

**DOI:** 10.1186/s40659-023-00424-7

**Published:** 2023-03-16

**Authors:** Ling Wang, Lei Wang, Meilian Tan, Linhai Wang, Wei Zhao, Jun You, Lijun Wang, Xingchu Yan, Wei Wang

**Affiliations:** 1 Key Laboratory of Biology and Genetic Improvement of Oil Crops of Ministry of Agriculture and Rural Affairs Oil Crops Research Institute of Chinese Academy of Agricultural Science, Wuhan, 430062 China; 2 Crop Institute, Gansu Academy of Agricultural Sciences, Lanzhou, 730070 China

**Keywords:** Drought stresses, Alternative splicing, DNA methylation, Linseed, Transcriptome

## Abstract

**Background:**

Drought stress has significantly hampered agricultural productivity worldwide and can also result in modifications to DNA methylation levels. However, the dynamics of DNA methylation and its association with the changes in gene transcription and alternative splicing (AS) under drought stress are unknown in linseed, which is frequently cultivated in arid and semiarid regions.

**Results:**

We analysed AS events and DNA methylation patterns in drought-tolerant (Z141) and drought-sensitive (NY-17) linseed under drought stress (DS) and repeated drought stress (RD) treatments. We found that the number of intron-retention (IR) and alternative 3’ splice site (Alt3’SS) events were significantly higher in Z141 and NY-17 under drought stress. We found that the linseed response to the DS treatment was mainly regulated by transcription, while the response to the RD treatment was coregulated by transcription and AS. Whole genome-wide DNA methylation analysis revealed that drought stress caused an increase in the overall methylation level of linseed. Although we did not observe any correlation between differentially methylated genes (DMGs) and differentially spliced genes (DSGs) in this study, we found that the DSGs whose gene body region was hypermethylated in Z141 and hypomethylated in NY-17 were enriched in abiotic stress response Gene Ontology (GO) terms. This finding implies that gene body methylation plays an important role in AS regulation in some specific genes.

**Conclusion:**

Our study is the first comprehensive genome-wide analysis of the relationship between linseed methylation changes and AS under drought and repeated drought stress. Our study revealed different interaction patterns between differentially expressed genes (DEGs) and DSGs under DS and RD treatments and differences between methylation and AS regulation in drought-tolerant and drought-sensitive linseed varieties. The findings will probably be of interest in the future. Our results provide interesting insights into the association between gene expression, AS, and DNA methylation in linseed under drought stress. Differences in these associations may account for the differences in linseed drought tolerance.

**Supplementary Information:**

The online version contains supplementary material available at 10.1186/s40659-023-00424-7.

## Background

Abiotic stresses such as drought, salinization, and excessive temperatures have severely hampered global agricultural productivity and are responsible for a 50%–70% drop in the production of the world’s principal crops [1]. As the global climate continues to change, drought has become the most significant unfavourable factor affecting plant growth, reducing crop yield more than any other abiotic stress [2]. Oil flax (*Linum usitatissimum* L.), also known as linseed is an important cash crop globally, and its products are used in industrial production, food processing, and cosmetics. Recent studies have shown that α-linolenic acid (ALA) and secoisolariciresinol diglucoside (SDG) in linseed oil can promote mammalian nervous system development and reduce the risk of breast cancer [3–6]. In addition, linseed has stronger drought stress tolerance than other cash crops and can grow in an arid environment with only 10% soil absolute water content [7]. Thus, it is widely grown in arid or semiarid areas such as India, Pakistan, and the northwest provinces of China, which face the highest drought frequency and have the longest recorded drought in East Asia [8, 9]. Recently, there have been some studies on linseed transcriptome analysis under different drought stresses; however, the molecular mechanism of linseed drought tolerance remains unclear [7, 10]. Therefore, understanding the complex responses of linseed to drought stress is essential in elucidating the mechanism of the response to drought stress in drought-tolerant crops and enhancing their yield and quality potential in breeding programs.

Alternative splicing (AS) is the process of processing the precursor mRNA (pre-mRNA) of a single gene at different splicing sites to produce multiple mature mRNA subtypes. It is a basic regulatory mechanism in plants that not only enhances the diversity of the plant transcriptome and proteome but also contributes to the specificity of gene function. AS events include intron retention (IR), mutually exclusive exons (MXE), exon skipping (ES), alternative 5’ splicing site (Alt5’SS), and alternative 3’ splicing sites (Alt3’SS) [11]. Among them, intron retention is the most common AS event in plants, and often produces some nonfunctional mRNAs, which are immediately degraded by nonsense-mediated decay (NMD) or by the production of truncated proteins [12–14]. In addition, AS may change the encoded protein structure and function by changing the gene sequence [15]. Although the function of most multiple-transcripts is poorly understood, well-studied cases demonstrate that AS is a key regulator in plant resistance to drought stress [16, 17]. *OsBWK1* is a member of the rice *MAPK* gene family, and it has three AS products, namely, *OsBWK1L*, *OsBWK1M*, and *OsBWK1LS*. The protein encoded by *OsBWMK1L* is transferred from the cytoplasm to the nucleus and participates in stress response regulation when rice is under abiotic stress [18]. Moreover, AS can also be involved in the plant response to abiotic stress by regulating transcription factors (TFs) such as *AP2*/*ERFBP*, *MYB*, *WRKY*, *NAC*, and *bZIP* when plants are under various abiotic stresses [19, 20].

Epigenetic modification can regulate gene expression without changing the DNA nucleotide sequence. Epigenetic modification includes DNA methylation, histone modification, and chromatin remodelling. These modifications affect the structure and accessibility of plant chromatin, thus regulating gene expression [21, 22]. Among them, DNA methylation is the most widely studied type of epigenetic modification, which usually occurs in CG, CHG, and CHH sites in plants [23]. Recent studies have shown that genome DNA methylation levels change under different environmental stresses, whether abiotic or biotic [24–27]. For example, the whole-genome DNA methylation level significantly increased after drought treatment in *Populus*, and many drought stress response gene expression patterns changed [28]. In addition, analysis of the differentially accumulated transcripts (DATs) of sesame led to the finding that due to changes in genome methylation levels under drought stress, the levels of 77% of DATs decreased, whereas at the recovery stage, the levels of more than 80% of DATs increased [29]. DNA demethylation also plays an important role in the plant response to drought stress through the involvement of plant abscisic acid (ABA) regulation [30].

Accurately identifying AS and DNA methylation sites requires analysing high-throughput sequencing data [31, 32]. However, second-generation sequencing (SGS) technologies have obvious limitations, especially as their short read lengths require computational assembly, making it difficult to infer the actual splice-site combination usage, which limits the accuracy of gene model predictions. With the development of high-throughput sequencing technology, single-molecule real-time (SMRT) sequencing offers much longer read lengths than SGS methods, thus eliminating the need for assembly and providing more direct evidence for the transcriptional isoforms of each gene [7, 33, 34]. However, SMRT has lower throughput and higher error rates, hence the advantages of SMRT sequencing and SGS are complementary.

Although previous studies have shown that transcriptional and posttranscriptional regulation may change in plants under drought stress [17], little is known about gene regulation at the posttranscriptional level in plants grown in arid or semiarid regions such as linseed. Therefore, in this study, we applied SMRT sequencing and SGS technologies to sequence the transcriptome of linseed. To simulate the effects of drought stress on linseed more accurately, we used repeated drought stress (RD) (including drought stress and rewatering) treatments to stimulate the response of linseed to different drought stresses. Subsequently, RNA-seq and BS-seq were used to analyse AS variants and DNA methylation changes and their interactions under drought stress in linseed seedlings with different drought tolerances. Considering the complex response of plants to drought stress, the physiological and transcriptional responses of leaves and roots to drought stress are almost entirely different [35, 36]. In this study, we only analysed and discussed the changes in alternative splicing and methylation in the aerial parts under drought stress to determine the molecular mechanism in response to drought stress. Our study revealed that both linseed varieties rapidly accumulated AS events under drought stress. Genome-wide methylation analysis revealed that a substantial number of hyper-DMRs in drought-tolerant linseed varieties under repeated drought stress, mainly concentrated at the CHH and CHG loci. In addition, our study also found that only the hypermethylated differentially spliced genes (DSGs) and the hypomethylated DSGs in the gene body region were enriched for plant abiotic stress response Gene Ontology (GO) terms in drought-tolerant and drought-sensitive linseed varieties, respectively.

## Materials and methods

### Plant materials, drought stress and RNA sequencing

RNA-Seq data used in this study were generated in our previous reports (NCBI SRA database: PRJNA598287) using the following procedure [7]. Drought-sensitive linseed variety NY-17 (accession no.: NYS-2005001), which is widely cultivated in China, was provided by the Guyuan Branch of the Ningxia Academy of Agriculture and Forestry Sciences, while drought-tolerant linseed variety Z141 (China metaphase germplasm bank no.: HM00001753), which was introduced from Alberta, Canada, was provided by the Zhangjiakou Academy of Agricultural Sciences.

The seeds of each linseed variety were randomly planted in 6 pots, 3 of which were randomly selected as the control group, with the other 3 pots as the experimental group. Six linseed seeds were planted in each pot, and there were 3 biological repeats for drought stress and the control. Drought stress (DS) and RD stress experiments began at 20 d after linseed germination. Subsequently, the soil water content of the experimental group was gradually reduced until the absolute soil water content (ASWC) was ~ 10%. Two days after maintaining ASWC at 10%, the phenotypic traits of the experimental group and the control group were measured. Subsequently, the stressed plants were watered to reach 70% ASWC, to help in recovery. The drought stress treatment was repeated when the ASWC was maintained at 70% for 5 days. Finally, irrigation was normally maintained until the maturation stage.

Total RNA from leaf tissues was extracted using TRIzol reagent (Invitrogen), according to the manufacturer’s instructions. mRNA was purified from the total RNA using poly Toligo-attached magnetic beads. Sequencing libraries were generated using the NEBNext® Ultra™ RNA Library Prep Kit for Illumina® (NEB, USA) following the manufacturer’s recommendations. The library quality was assessed on the Agilent Bioanalyzer 2100 system. Each SMRT cell line was sequenced using P6 C4 reagent on the PacBio RS II platform with 4 h sequencing movies. After processing raw data, we obtained more than 33 million filtered subreads with a mean length of ~ 2000 bp (Additional file 1).

### Belowground biomass dry weight measurement

The roots of Z141 and NY-17 after the DS and RD treatment were removed from the nutrient soil (ratio of cultivating soil and vermiculite was 1:1) and rinsed clean. Then each root was placed in an aluminium box individually and dried in an oven at 80 ℃ to constant weight. Finally, the dry weight of each root was measured.

### AS detection and drought stress-responsive AS event identification

First, we used fastp (*ver.* 0.21.0) software to filter some low-quality reads in the original sequencing reads (for example, the average quality value of the whole reads is lower than 20) and remove the adapters in some of the reads [37]. Then the cleaned reads were aligned to the linseed reference genome (assembly version: BGIv1.0, https://phytozome.jgi.doe.gov/pz/portal.html#!info?alias=Org_Lusitatissimum) using HISAT2 (*ver.* 2.1.0), and only uniquely aligned reads were retained for subsequent analysis [38]. The AS events were identified using rMATS (*ver.* 4.1.2) software. To more accurately identify AS events, we used annotation files that were reoptimized after three generations of SMRT transcriptome sequencing, and set the main parameters as -t paired –readLength 150 –novelSS –tstat 4 –variable-read-length [39].

Drought stress-responsive AS event identification in the linseed genome under DS and RD treatments was performed using rMATS (*ver.* 4.1.2) software comparing bam files under DS or RD treatments and controls. To accurately identify drought stress-responsive AS events, we set FDR < 0.05 and IEP > 0.2 [40].

### Cluster analysis of drought stress-responsive AS genes

To further clarify the expression pattern of the drought stress response of AS events under different drought stresses, we subsequently used a self-written script program to remove the duplicate AS events and then count the expression level (ΔIEP value) of each AS event in response to drought stress. Finally, the R program Mfuzz (*ver.* 2.4.8.0) was used to cluster the drought stress-responsive AS events in Z141 and NY-17 according to the AS event expression level change under the DS, rewatering (RW), and RD treatments [41].

### MethylC-seq library preparation and analysis

DNA was extracted using the CTAB method, and the specific experimental steps were as described by Clarke [42]. Bisulfite-treated linseed DNA samples were used to prepare MethylC-seq libraries. A total of 24 DNA samples (1 control and 3 different drought treatments × 3 biological × 2 linseed varieties) were sent to BioMarker Biotechnology Co., Ltd. (Beijing) for sequencing library construction and methylC-sequencing. Sequencing libraries were prepared using a NEXTFLEX™ Bisulfite Sequencing Kit (PerkinElmer, USA) following the manufacturer’s instructions, followed by MethylC- sequencing using the Illumina NovaSeq 6000 platform (San Diego, USA).

Quality assessment of the sequenced reads and low-quality reads and adapter deletion were performed using fastp (*ver.* 0.21.0) software, followed by mapping of the remaining high-quality reads to the Lusitatissimum reference genome version (BGIv1.0) using the Bismark (*ver.* 0.23.0) software [43]. All methylated cytosines were identified using R package methylKit for each of the 24 samples and only considered if cytosines were covered by at least 5 high-quality reads [44]. Three methylation call files were generated for each drought stress treatment, corresponding to the CG, CHG, and CHH sequence contexts. Then, the reference genome was divided into nonoverlapping windows of 200 bp, and the R package methylKit was used to identify the hyper- and hypomethylated regions (200 bp bins) of Z141 and NY-17 in response to drought stress. Among them, the minimum methylation difference of CG, CHG, and CHH sequences was 50%, and FDR < 5%.

### GO and REVIGO enrichment analysis

For the GO enrichment analysis in this study, we used the R package clusterProfiler [45]. To make the GO results more intuitive, we used REVIGO (http://revigo.irb.hr/) to summarize the GO terms with significant GO enrichment (*P* ≤ 0.05) and created a treemap [46].

### Statistical analysis

For all comparisons involving pairs of means, we used independent t tests. Statistical analysis was performed using SPSS ver. 21.0 software package for Windows (IBM Inc., New York, USA).

## Results

### IR is the most frequent type of AS event in linseed

**Fig. 1:**
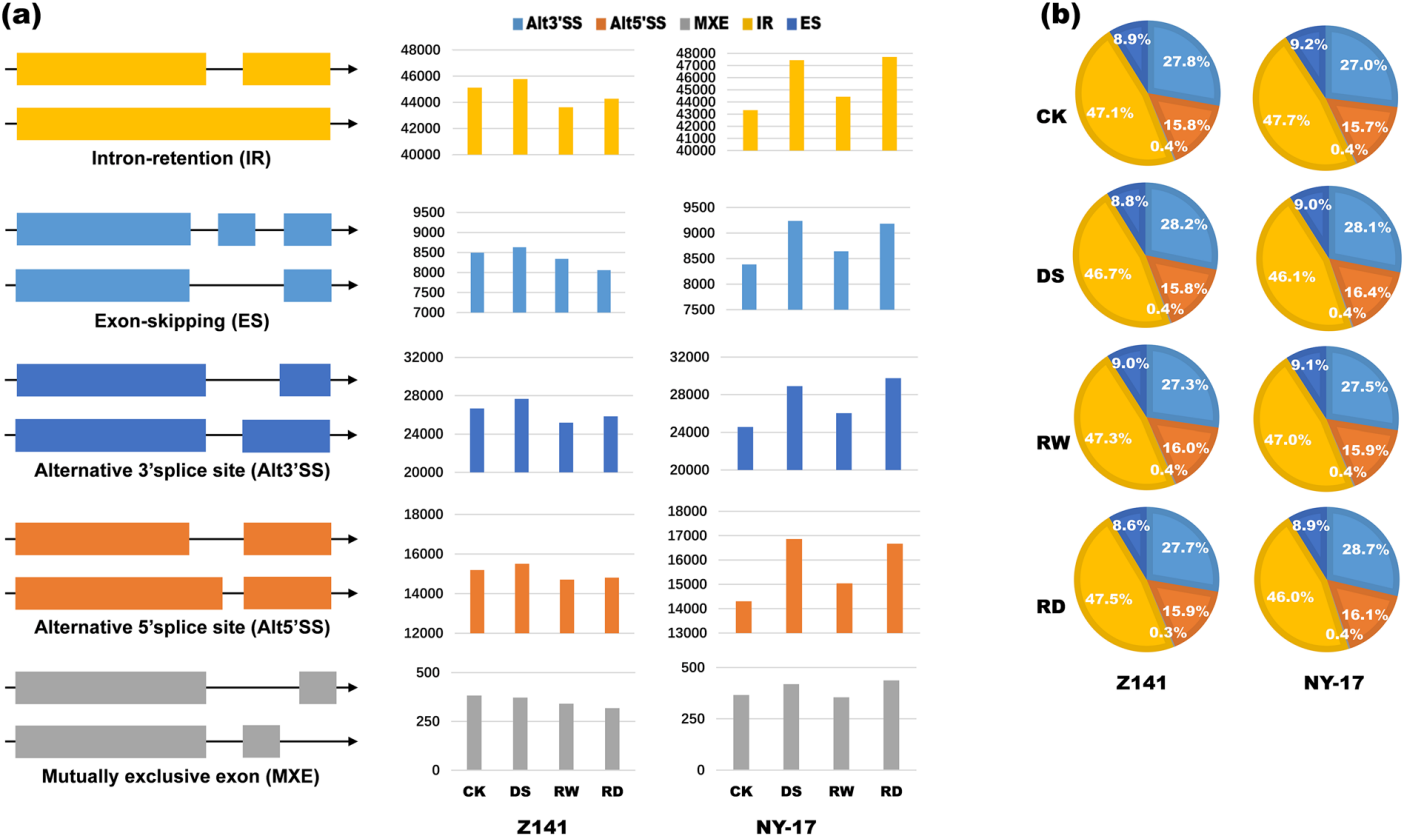
Summary of alternative splicing (AS) events in linseed seedlings. **a** Schematic diagram of five alternative splices and their numbers in Z141 and NY-17. Yellow, light blue, dark blue, orange and grey indicate intron retention (IR), exon skipping (ES), alternative 3′ splicing sites (Alt3′SS), alternative 5′ splicing sites (Alt5′SS) and mutually exclusive exons (MXE), respectively. The right bar graph depicts the number of corresponding AS events in Z141 and NY-17. The ordinate indicates the number of AS events, and the abscissa indicates the control check (CK), drought stress (DS), rewatering (RW) and repeated drought stress (RD) treatments. **b** Distribution of AS events in Z141 and NY-17 under different treatments

To investigate the transcriptome response to different degrees of drought stress, we performed SMRT sequencing on linseed seedlings under the DS, RW, and RD. Our previous study showed that more than 1,000 different functional genes closely respond to drought stress, with their expression patterns changing with drought stress [7]. Therefore, in this study, we used the same sequencing data for comprehensive profiling of the AS landscape of linseed seedlings under the DS and RD treatments (Additional file 1). In this study, a total of 146,071 AS events were identified in the drought-tolerant linseed variety Z141 after drought stress, corresponding to 19,062 genes, or 50.52% of all expressed genes. Among them, 16,536, 16,171, and 16,309 AS genes were determined in DS, RW, and RD, respectively, accounting for 46.52%, 45.08%, and 45.71% of the expressed genes (Fig. 1a, Additional files 2 and 3). In the drought-sensitive linseed variety NY-17, we identified 156,843 AS events after drought stress and corresponding to 19,425 genes, accounting for 51.14% of all expressed genes. We identified 16,780, 16,316, and 16,878 AS genes in the DS, RW, and RD treatments, respectively, which accounted for 46.97%, 45.61%, and 46.68% of all expressed genes in linseed seedlings (Fig. 1a, Additional files 2 and 3). Furthermore, although the number of AS events in Z141 and NY-17 differed under drought stress, the distribution of AS types was consistent. The concrete manifestation is that IR was the most abundant AS event (46% ~ 48%), followed by Alt3’SS (27% ~ 29%), Alt5’SS (15% ~ 16%), ES (8% ~ 9%), and MXE (≤ 1%) (Fig. 1b).

Notably, the AS pattern changes between Z141 and NY-17 induced by DS and RD treatment were inconsistent. Specifically, both Z141 and NY-17 accumulated a large number of IR events when they were first exposed to drought stress. However, when Z141 and NY-17 were exposed to repeated drought stress, the AS pattern change in NY-17 was similar to that of the first exposure to drought stress, with a large number of accumulated IR events corresponding to repeated drought stress. (Fig. 1a).

### Differential alternative splicing of linseed genes induced by drought stress

**Fig. 2:**
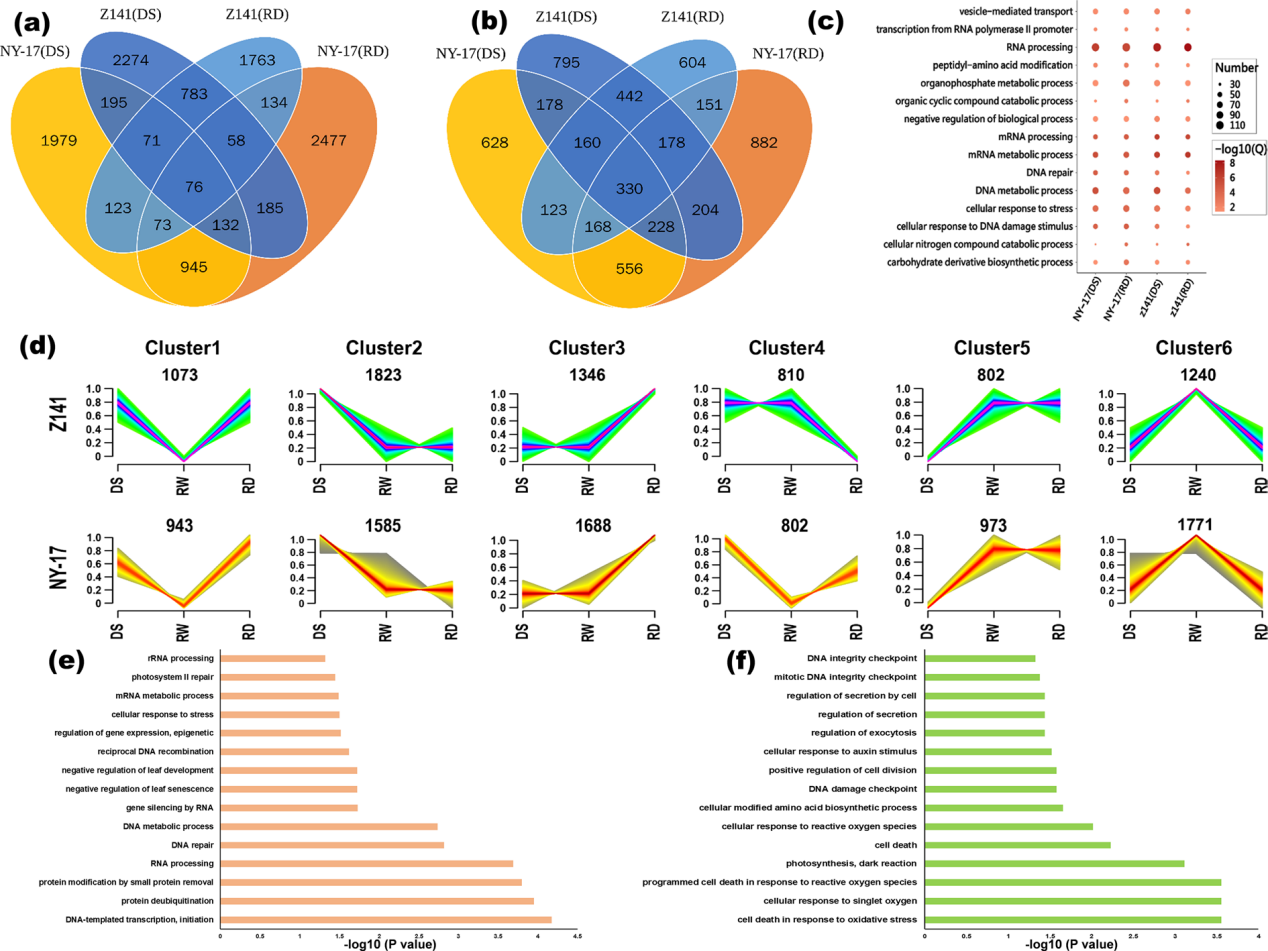
Identification of drought stress-responsive AS events in linseed seedlings. **a** The number of drought stress-responsive AS events identified in Z141 and NY-17 under the DS and RD treatments. **b** The number of differentially spliced genes (DSGs) identified in Z141 and NY-17 under the DS and RD treatments. **c** Bubble plots showing examples of Z141 and NY-17 GO enrichment under different drought stresses. the bubble size indicates the number of enriched genes. The complete GO analysis results are provided in Additional file 5. **d** Clustering analysis of drought stress-responsive AS events was performed based on changes in isoform expression percentage (IEP) after the DS, RW and RD treatments. According to the changes in IEP between the control and each stress condition, the drought stress responses to AS events were clustered into 6 groups. The numbers in the figure indicate the number of AS events for the corresponding cluster. The abscissa indicates different drought stress treatments, and the ordinate indicates the normalized ΔIEP value. The darker line in each graph indicates the average expression trend. **e**, **f** GO terms significantly enriched by DSGs of Z141**e** and NY-17 **f** in Cluster 1

In this study, stress-responsive AS events were defined by criteria junction reads based on FDR < 0.05 and ≥ 20% variation in InclevelDifference (calculated as lncLevel1—lncLevel2) change after stress treatments. Finally, 5,867 and 6,448 drought-responsive AS events were identified in Z141 and NY-17, respectively (Fig. 2a, Additional file 4). These AS events were located in 3,561 and 3,786 protein-coding genes in Z141 and NY-17, respectively (Fig. 2b, Additional file 4). Then, we counted the AS events and DSGs of Z141 and NY-17 in response to the DS and RD treatments. The results are shown in Fig. 2a, b; 3,594 and 4,080 differential AS events located in 2,371 and 2,697 DSGs were identified in NY-17. In Z141, 3,774 and 3,081 differential AS events were identified, and these AS events were located in 3,588 and 2,165 DSGs under the DS and RD treatments, respectively. To determine the gene function of these DSGs, we performed GO enrichment analysis for genes regulated by AS under DS in NY-17 and Z141. The results are shown in Fig. 2c and Additional file 5. In Z141 and NY-17, the genes that were enriched in abiotic stress-related GO terms such as DNA repair, cellular response to stress, and cellular response to DNA damage stimulus were regulated by AS. Interestingly, our data showed that these drought stress response AS events were highly cultivar specific. Specifically, 474 and 371 overlapping drought stress response genes were identified in Z141 and NY-17 under the DS and RD treatments, respectively. These overlapping AS events only accounted for 10% or less of drought stress response AS events in Z141 or NY-17 under the DS or RD treatment (Fig. 2a). Functional analysis of these nonoverlapping genes showed that DSGs in Z141 and NY-17 were significantly enriched in abiotic stress response GO terms (e.g., ‘RNA splicing’, ‘response to water’, ‘cell response to stress’) and in specific GO terms (e.g., “root system development” in Z141 under the RD treatment) (Additional file 6). Our results indicated that drought stress-induced AS pattern changes were significantly different among different drought tolerant linseed varieties.

Subsequently, we performed cluster analysis on all differential splicing events to distinguish AS events that respond to different drought stress treatments. The result are shown in Fig. 2d. These AS events can be roughly divided into 6 categories based on the changing pattern of isoform expression percentage (IEP) under different stress treatments. Cluster 1, consisting of 1,073 (Z141) and 943 (NY-17) AS events, showed significant AS pattern changes under both the DS and RD treatments. Clusters 2–5, consisting of 4,781 (Z141) and 5,048 (NY-17) AS events, indicated significant AS pattern changes in response to the DS or RD treatments. Cluster 6 consisted of 1,240 (Z141) and 1,771 (NY-17) AS events, representing significant AS pattern changes only under the RW treatment. Notably, Groups 2 and 3 exhibited significant AS pattern changes only in the DS or RD treatments, which suggests that the DS and RD treatments can induce specific AS responses (Fig. 2d). Next, we analysed the gene functions in different clusters. Unsurprisingly, the GO enrichment results in the same cluster between Z141 and NY-17 showed almost no overlap (Fig. 2e, f, Additional file 7). For example, in Z141, the cluster1 genes were significantly enriched in “photosystem II repair”, “cellular response to stress”, “gene silencing by RNA”, “DNA repair” and other GO terms related to the plant response to abiotic stress (Fig. 2e). However, in NY-17, the cluster1 genes were significantly enriched in GO terms related to plant ageing such as "cell death", “programmed cell death in response to reactive oxygen species”, and “cell death in response to oxidative stress” (Fig. 2f). These results indicated that drought stress induced AS events in Z141 and NY-17 were significantly specific.

Studies have shown that Ser/Arg-rich (SR) proteins are key regulators of AS in plants [47]. Therefore, we analysed the gene expression and alternative splicing changes in Z141 and NY-17 SR genes in response to the DS, RW, and RD treatments. In Z141, we found that 21, 14, and 27 SR genes were regulated by differential splicing under the DS, RW, and RD treatments, respectively. In NY-17, 26, 13, and 29 SR genes were also regulated by differential splicing under the DS, RW, and RD treatments (Additional file 8). The differential expression analysis showed that compared with RW, more SR genes were upregulated under the DS and RD treatments. Unexpectedly, the expression levels of almost all SR genes were upregulated under the RD treatment in both Z141 and NY-17 (Additional file 8).

### Differentially expressed and alternatively spliced genes are coregulated in response to repeated drought stress

**Fig. 3:**
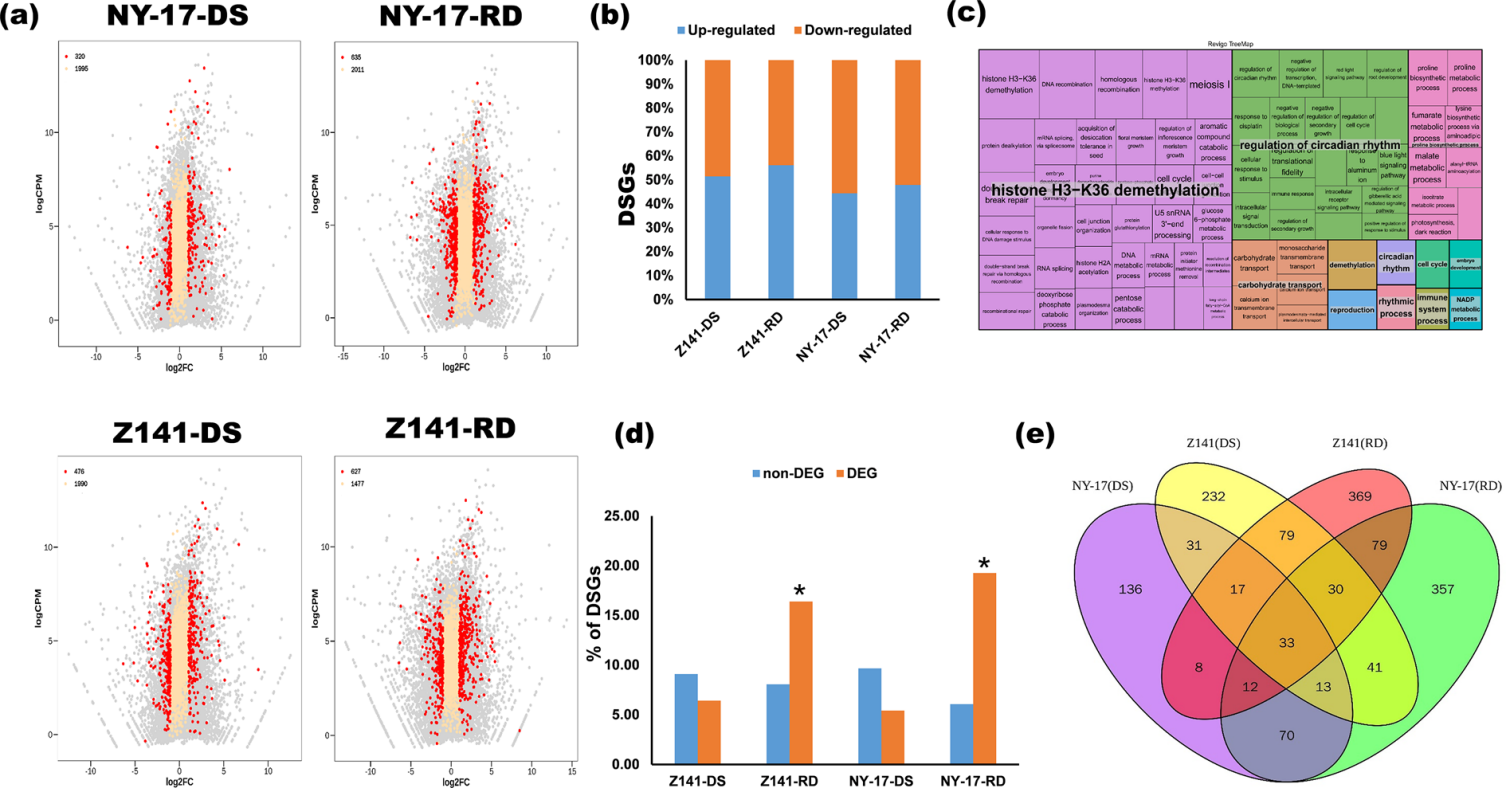
Comparison analysis of differentially spliced genes (DSGs) and differentially expressed genes (DEGs) in response to the DS and RD treatments in linseed seedlings. **a** Scatter plot showing AS patterns and gene expression changes after the DS and RD treatments. The abscissa indicates the fold of the difference in gene expression level, which is represented by the log2-transformed fold change (FC). The ordinate indicates the gene expression amount expressed by log-transformed counts per million reads (CPM). The red dots in the figure represent genes identified as both DSGs and DEGs, the yellow dots are genes identified as DSGs, and the grey dots represent genes identified as other expression genes. The numbers in the figure indicating the number of corresponding genes. **b** Histograms indicate the number of DSGs with upregulated (blue squares) and downregulated (orange squares) gene expression under the DS and RD treatments in Z141 and NY-17. **c** Tree diagram showing an example of the REVIGO analysis of overlapping DSGs and DEGs. The complete REVIGO analysis results are provided in Additional file 9. **d** Comparison of the proportion of DSGs identified in DEGs and non-DEGs under the DS and RD treatments. The proportion of DSGs in DEGs was much higher than that in non-DEGs in Z141 and NY-17 under the RD treatment, and the P values indicate significance levels based on Fisher’s exact test. **e** Venn diagram showing overlapping DSGs in Z141 and NY-17 under DS and RD treatments

To understand the relationship between transcriptome regulation and AS in different drought-tolerant linseed seedlings, we combined the analyses of the DSGs and DEGs in Z141 and NY-17 to elucidate their relationship in response to different drought stresses. The results are shown in Fig. 3a. For Z141 and NY-17, we identified 476 and 320 DSGs showing differential expression patterns under the DS treatment (indicated by red dots in Fig. 3a), accounting for 16% and 24% of all DSGs, respectively. Under the RD treatment, more DSG expression patterns changed, and we identified 635 and 627 DSGs showing differential expression patterns in Z141 and NY-17, accounting for 32% and 42% of all DSGs, respectively (Fig. 3a). To clarify whether up- or downregulated DEGs are regulated by AS to the same extent, we analysed the gene expression pattern changes in these DSGs. Our data showed that there was no significant difference in the proportion of DSGs that were up- or downregulated in Z141 and NY-17 under the DS and RD treatments (Fig. 3b). This result indicated that the up- or downregulated DEGs in this study were all regulated by AS to the same extent.

Using a simple clustering method that focuses on semantic similarity metrics, REVIGO can summarize and illustrate a vast and incomprehensible list of GO terms. [48]. Therefore, we used REVIGO in this study to analyse the biological functions of these DSGs. The results are shown in Fig. 3d; the functions of the DSGs whose expression levels are upregulated under the DS treatment in Z141 and NY-17 are mainly concentrated on epigenetic modifications (such as histone methylation/demethylation), regulation of circadian rhythm, proline biosynthesis process, and other GO terms related to plant response to abiotic stress [49, 50] (Fig. 3c and Additional file 9). The functions of DSGsthat were significantly upregulated under the RD treatment in Z141 and NY-17 were mainly enriched in proline biosynthesis (Additional file 9).

To examine whether AS changes in linseed under the DS and RD treatments can be affected by transcriptional activity, we compared the proportion of DSGs in the differentially expressed genes and non-differentially expressed genes in Z141 and NY-17. Our data showed that the proportion of DSGs in DEGs was significantly higher than that in non-DEGs (16% vs. 8% in Z141, 19% vs. 6% in NY-17) in Z141 and NY-17 under the RD treatment (Fig. 3d). However, no such difference was found for Z141 and NY-17 under the DS treatment (Fig. 3d). Subsequently, we also analysed the global IEP changes in AS in Z141 and NY-17 between DEGs and non-DEGs under the DS, RW, and RD treatments. Our results showed that the global IEP change in AS under the RD treatment was more intense in DEGs than in non-DEGs (Additional file 10). This result suggests that AS pattern changes may be associated with transcriptional activity under the RD treatment. However, no such difference was found in Z141 and NY-17 under the DS treatment. This result suggests that the association between the AS response and transcriptional activity may vary with different drought treatments.

To understand whether these DSGs have specificity, we compared the overlapping proportions of DSGs with gene expression changes between Z141 and NY-17. Our data showed that these DSGs did not exhibit any significant specificity. For example, 104 overlapping DSGs were identified between Z141 and NY-17 under the DS treatment. These DSGs accounted for 40% of the DSGs whose gene expression levels changed under the DS treatment (Fig. 3e).

Interestingly, we found that some DSGs whose gene expression was upregulated such as *AGB1*, *SNRK1*, and *AMP1*, were only identified in Z141 under the RD treatment (Additional file 11). These genes were reported to be associated with plant root development [51–53]. Therefore, we compared the differences in root growth between Z141 and NY-17 under the DS and RD treatments by measuring the dry weight of the belowground biomass. The results are shown in Table 1. Under the DS and RD treatments, the root dry weight of Z141 was 9% and 46% lower than that of the control, respectively, while the root dry weight of NY-17 was reduced by 34% and 68%, respectively. This result indicated that these genes regulated by transcription and AS may play a very important role in the plant response to drought stress.

**Table 1:** Belowground biomass dry weight of Z141 and NY-17 under drought treatments

Traits		Z141	NY-17
	CK	0.021 ± 0.0009	0.053 ± 0.008
Below-ground biomass dry weight (g)	DS	0.019 ± 0.0004	0.035 ± 0.006^**^
	CK	0.12 ± 0.03	0.18 ± 0.06
	RD	0.065 ± 0.004^*^	0.059 ± 0.007^***^

^*^p < 0.05

^**^p < 0.01

^***^p < 0.001

### Comparative analysis of the biological functions regulated at the AS and transcription levels

**Fig. 4:**
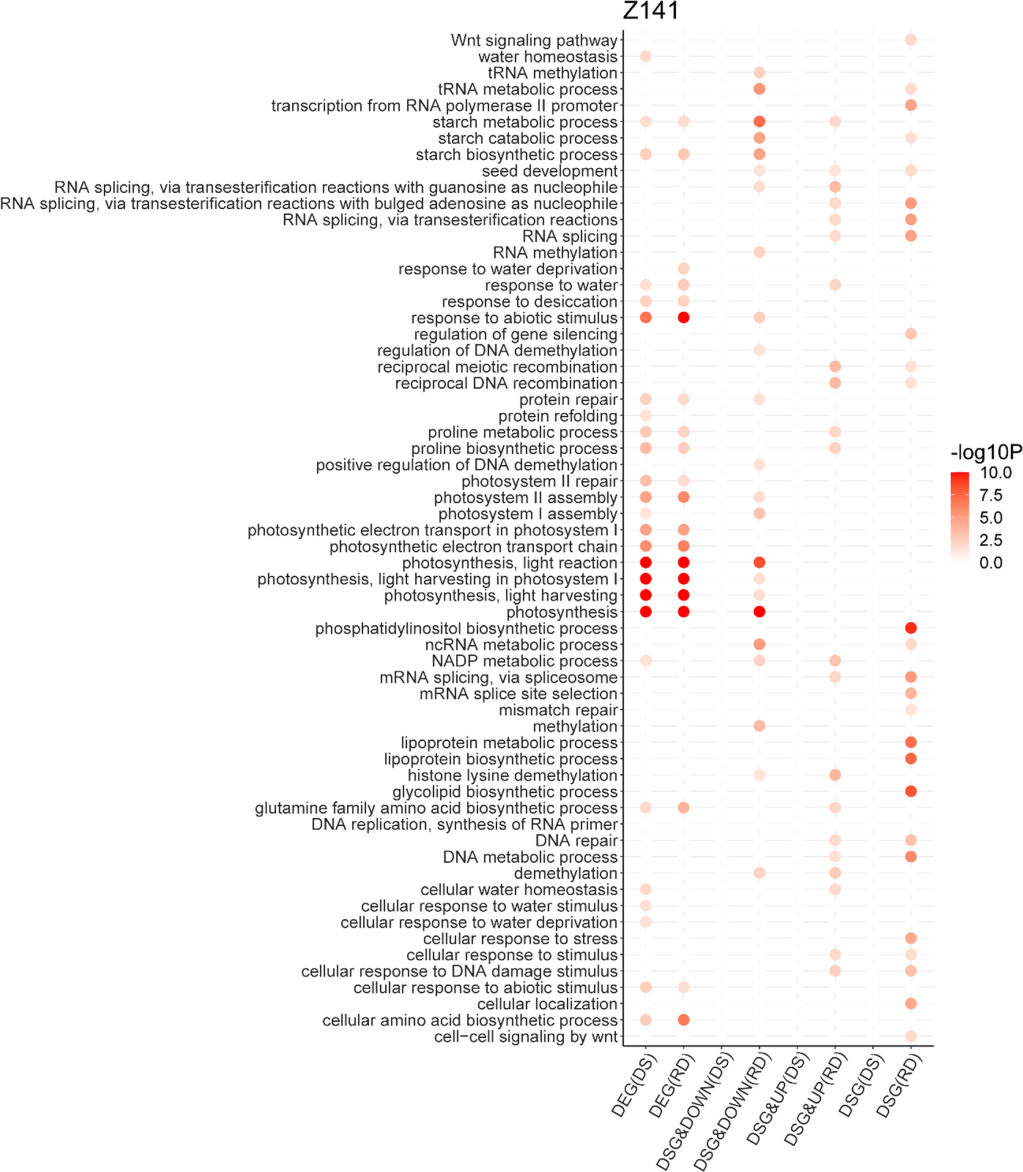
Functional enrichment analysis of DSG-specific, DEG-specific and overlapping DEGs&DSGs in Z141. The enriched Gene Ontology (GO) terms of DSG-specific, DEG-specific and overlapping DEGs&DSGs are shown in a bubble diagram. DSG: DSG-specific genes; DSG&Up: genes identified to be both differentially spliced and upregulated; DSG&Down: genes identified to be both differentially spliced and downregulated; DEG: DEG-specific genes; DS: drought stress treatment; RD: repeated drought stress treatment

To clarify the biological functions of genes regulated by AS and/or transcriptionally, we performed GO enrichment analysis for DSG-specific, DEG-specific, and overlapping DEGs&DSGs. Our results showed that very few DSG-specific genes were significantly enriched in drought stress response GO terms under the DS treatment, while DEG-specific genes were significantly enriched in various drought stress response GO terms, such as “photosynthesis”, “response to abiotic stress”, and “proline biosynthesis” (Fig. 4, Additional file 12). This result indicated that the linseed response to the DS treatment was mainly regulated at the transcriptional level, which was consistent with our observations. However, in Z141 and NY-17 under the RD treatment, we found that some abiotic stress response GO terms, including "DNA repair", "photosynthesis", "cellular response to DNA damage stimulus", and "proline biosynthesis" were significantly enriched in overlapping DEGs&DSGs (Additional file 12). In addition, we also found that a group of epigenetics GO terms (e.g., “demethylation”, “RNA methylation”, “histone lysine demethylation”) was significantly enriched in overlapping DEGs&DSGs under the RD treatment. Collectively, our results demonstrate that a wide range of abiotic stress-responsive pathways were dually regulated at both the transcriptional and AS levelsin Z141 and NY-17 in response to the RD treatment.

### Drought stress induces a slight increase in the global DNA methylation level in linseed

**Fig. 5:**
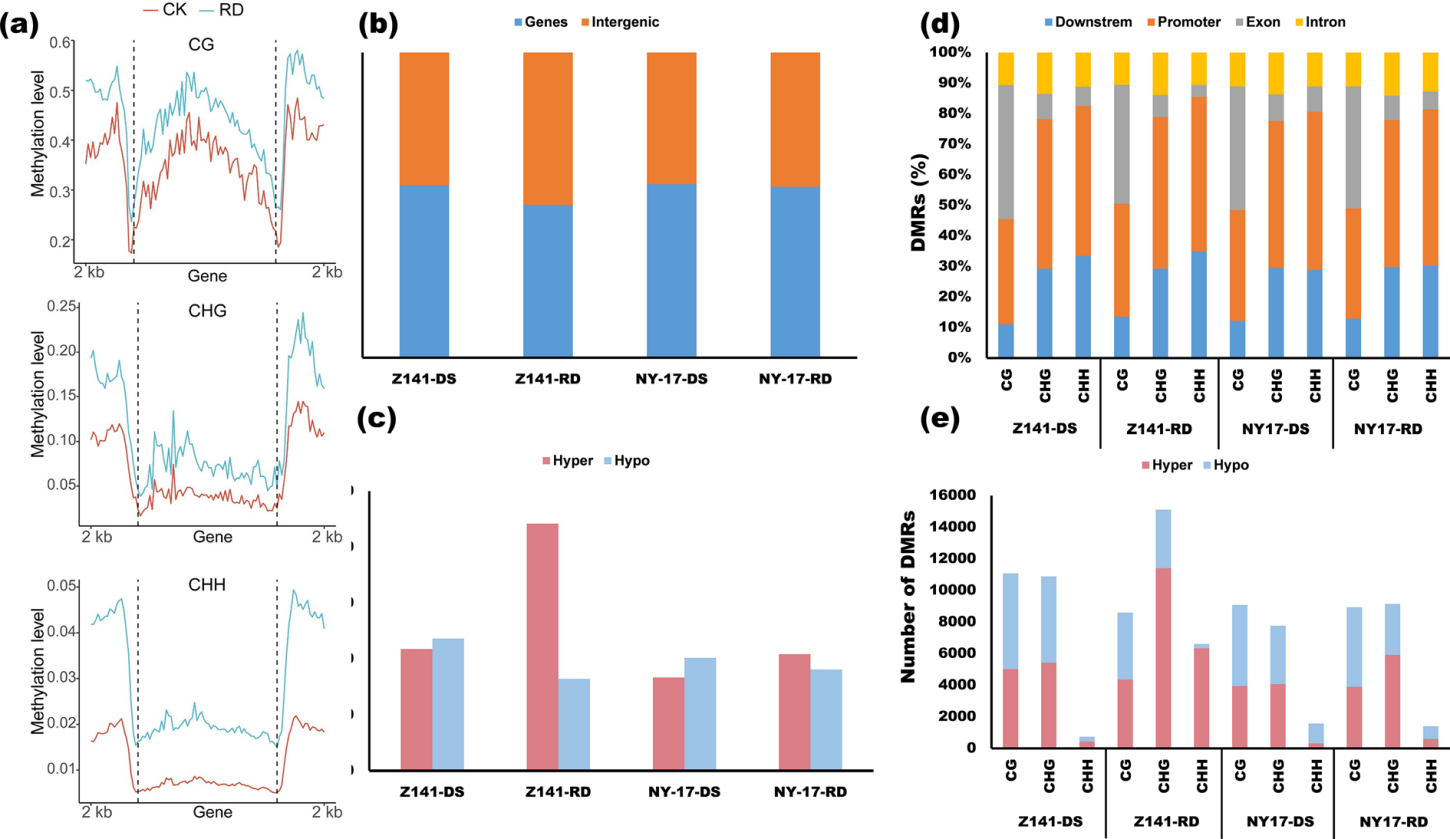
Methylome landscape of linseed under the DS and RD treatments. **a** An example of a comparison of global DNA methylation levels. The figure indicates a comparison of global DNA methylation levels between the RD treatment (blue) and CK (red) over protein-coding genes of Z141 under the RD treatment in the context of CG, CHG, and CHH sequences. The complete results are provided in Supplementary Table S8. **b** The percentage of differentially methylated regions (DMRs) in the gene body region and the intergenic region. (**c**) The total number of hyper and hypo-DMRs identified under the DS and RD treatments. **d** The percentage of DMRs overlapping with different features of protein-coding genes, including promoters, exons, introns, and downstream region. **e** DNA methylation sequence contexts of DMRs identified in linseed under the DS and RD treatments

Drought stress often alters plant DNA methylation levels [54]. Our previous analysis results also showed that the key genes involved in DNA methylation and active demethylation (e.g., *DRM2* and *MBD7*) were significantly upregulated in linseed seedling tissues compared with CK under drought stress (Additional file 13). Therefore, we used bisulfite sequencing to analyse the genome-wide DNA methylation of linseed seedlings under two different drought stress treatments using the same tissue used for RNA sequencing. Analysis of the global DNA methylation of linseed seedlings showed that the cytosine methylation level was higher than that of the control materials (CK) in CG, CHG, and CHH during the DS and RD treatment stages (Fig. 5a and Additional file 13).

To further illustrate the differences in DNA methylation, we identified differentially methylated regions (DMRs) of linseed seedling leaf tissues between drought stress and CK using an FDR of less than 0.01 and at minimum methylation difference values of CG, CHH, and CHG of 0.5, 0.2, and 0.1, respectively. In this study, 22,705 and 18,433 DMRs were identified in Z141 and NY-17 under the DS treatment, respectively (Additional file 14), of which approximately 43% of the DMRs were mapped in unannotated genomic regions and approximately 57% of the DMRs mapped in protein-coding genes (Fig. 5b). Under the RD treatment, we identified 30,315 and 19,481 DMRs in Z141 and NY-17, respectively (Additional file 14). Next, we counted the number of hyper and hypo-DMRs. The results are shown in Fig. 5c. More hypo-DMRs were identified in Z141 and NY-17 under the DS treatment, while more hyper-DMRs were identified under the RD treatment (Fig. 5c).

To understand the distribution of three methylated contexts (CG/CHH/CHG) on expressed genes, we mapped DMRs to annotated protein-coding genes. The results revealed that the DMRs at CG sites were mainly mapped in the exon and promoter region, and more than 50% of the DMRs at CHH and CHG sites were mapped in the promoter region (Fig. 5d). Furthermore, we analysed the DMR distribution in genes in Z141 and NY-17 under the DS and RD treatments. We found that DMRs in the contexts of CG and CHG were more abundant than CHH-DMRs (Fig. 5e). In addition, the number of CHH-DMRs and CHG-DMRs were higher in Z141 and NY-17 under the RD treatment; specifically, the number of hyper CHH-DMRs and hyper CHG-DMRs were most significantly higher (Fig. 5e).

### Gene body methylation dynamics are not associated with differential RNA splicing

**Fig. 6:**
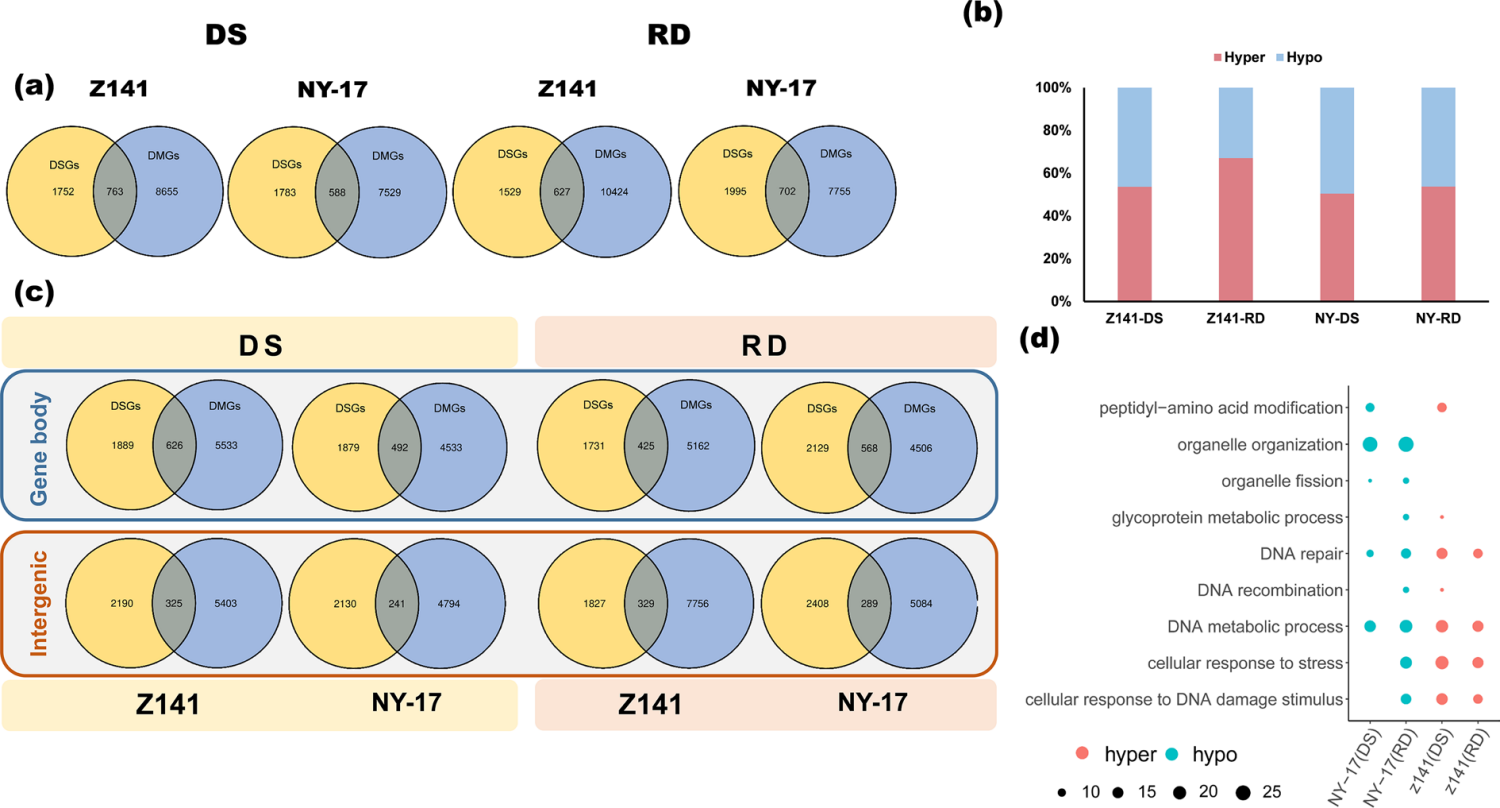
Comparison analysis of differentially methylated genes (DMGs) and differentially spliced genes (DSGs) in response to the DS and RD treatments in linseed seedlings. **a** Venn diagram showing the number of overlapping DSGs and DMGs in Z141 and NY-17 under the DS and RD treatments. **b** Stacked bar graphs showing the number of DMGs and DSGs in gene body regions and intergenic regions. Blue, orange and grey indicate DMGs, overlapping DMGs and DSGs, and DSGs, respectively. The numbers in the figure indicate the number of corresponding genes. **c** Venn diagrams indicating the number of DSGs with methylation level changes in the gene body and the intergenic region. **d** Bubble plots showing examples of GO enrichment of overlapping DSGs and DMGs in Z141 and NY-17. The bubble size indicates the number of enriched genes, the red bubbles indicate hypermethylation, and the blue bubbles indicate hypomethylation

To clarify the relationship between methylation and differential RNA splicing in linseed under different drought stresses, we first counted the number of overlapping DMGs and DSGs in Z141 and NY-17. The results are shown in Fig. 6a. Under the DS and RD treatments, 763 and 627 DSGs were identified as overlapping DSGs and DMGs in Z141, accounting for 30% and 29% of all DSGs, respectively (Fig. 6a). In NY-17, we identified 588 and 702 overlapping DMGs and DSGs, accounting for 25% and 26% of all DSGs, respectively (Fig. 6a). Subsequently, we analysed the proportion of hyper-DMR and hypo-DMR in these DSGs. In most cases, these DMRs did not show a hypo-/hyper bias (Fig. 6b) Further analysis showed that DMRs in intergenomic regions (including promoter regions and downstream regions) were identified in only approximately 10% of DSGs (Fig. 6c). Considering that this includes genes with DMRs identified in both the gene body region and the intergenic region, the proportion of DSGs in which the methylation level only changed in the intergenomic region should be lower. Therefore, we focused on the biological function of those DSGs. However, GO enrichment analysis yielded unexpected results. We observed that only overlapping hyper-DMGs and DSGs in Z141 under the DS and RD treatments were significantly enriched in GO terms related to abiotic stress responses (for example, "cellular responses to stress" and "cellular responses to DNA-damaging stimuli"), whereas in NY-17, only overlapping hypo-DMGs and DSGs were enriched in these GO terms (Fig. 6d).

However, we assessed the relationship between DNA methylation changes and DAS using the method described by Harris et al. (2019), which correlates the level of differential methylation on DAS exons with differences in PSI. We found no correlation between methylation differences and ΔPSI (Additional file 15), suggesting that changes in methylation status may not be related to changes in DAS events induced under the DS or RD treatments in linseed.

## Discussion

Drought stress has become the most significant environmental stress affecting global agriculture as a result of climate change and a growing global population, which have led to a shortage of freshwater resources. The inability of plants to actively avoid drought stress necessitates the evolution of intricate systems at the transcriptional and posttranscriptional levels. In recent years, some studies have investigated the changes in transcriptional and posttranscriptional regulation in plants under drought stress, such as wheat, tea, and soybean [17, 55, 56], yet gene regulation at the posttranscriptional level in plants that are cultivated in arid or semiarid regions (e.g., linseed) is less known. AS, as a posttranscriptional regulation, plays an important role in plant defence against abiotic stress [15, 57]; however, the contribution of AS to drought tolerance in linseed remains unclear. In this study, we identified genome-wide AS events in linseed seedlings under different drought stresses and revealed the AS profiles in response to different drought stresses.

IR events are an important type of AS, and stress-induced IR is a common phenomenon in plants [58, 59]. We obtained similar results in this study; more than 140,000 AS events were identified in Z141 and NY-17, of which more than 46% were IR events (Fig. 1b, Additional file 2 and Additional file 3). The number of IR events in Z141 and NY-17 increased rapidly under drought stress and decreased rapidly after rewatering (Fig. 1a). Not surprisingly, the transcripts of some key genes that have been demonstrated to respond positively to drought stress contain IR events. For example, *P5CS* is a key gene in proline biosynthesis and plays an important role in plant resistance to drought and saline-alkali stress [7]. In this study, we identified IR events in the transcripts of some members of the *P5CS* family in Z141 and NY-17 under drought stress (Additional file 4). Similar results were also found in salt-stressed cotton [60]. IR events in the transcripts of some genes that may be related to the plant response to abiotic stress, such as *ARP* and *CRY*, have been identified [61, 62] (Additional file 4). Furthermore, in this study, we also demonstrated that drought stress and repeated drought stress had a reduced effect on the root growth of Z141 (Table 1). The genes (such as *AGB1*, *SNRK1*, and *AMP1*) that have been reported to promote plant root growth and development [51, 63, 64] were also identified as IR events in the transcriptome of Z141 in this study (Additional file 4). Overall, our findings suggest that IR plays a key role in regulating the linseed response to drought stress by diversifying transcriptome reprogramming, but its biological relevance requires further experimental investigation.

To date, the molecular mechanisms of the plant AS response to drought stress are still largely ambiguous. A large number of studies have shown that the precision and efficiency of pre-mRNA splicing require the cooperation of cis-splicing elements and splicing-related proteins [65]. Serine/arginine-rich (SR) proteins, as important plant AS regulators, participate in a large number of plant AS pattern changes [47, 66]. Studies have shown that the richness of UA in introns and CG in exons contributes to the splice-site recognition of Ser/Arg-rich (SR) genes [17]. The abundance and activity of SR genes are affected by AS pattern changes, which are affected by abiotic stresses, including drought stress [67]. In this study, the number of SR genes that responded to the DS and RD treatments was greater than that under the RW treatment, at both the transcriptional and splicing levels (Additional file 7). This result might explain why there are more splice variants under the DS and RD treatments.

A transcriptome study revealed differences in drought-induced AS patterns between drought-tolerant and drought-sensitive rice cultivars and suggested that these differences may be an important strategy for drought resistance in upland rice during long-term domestication [68]. However, other studies have shown that drought stress induces a reduction in the number of AS events in drought-tolerant cultivars [69]. This finding indicates that drought stress has different effects on AS patterns in plants with different drought tolerances. Accumulating experimental evidence suggests that higher stress-induced AS in genotypes is not necessarily associated with stress tolerance, but the frequency of genotype-specific AS events, the abundance of AS transcripts associated with key stress-response processes, and the nature of the splicing events may contribute to stress tolerance [70]. Consistently, in this study, we found that the number of differential AS events responding to the DS or RD treatment was not different between Z141 and NY-17, but the proportion of overlapping differential AS events between Z141 and NY-17 under the same treatment was low, i.e., 10% (Fig. 2a). This finding is consistent with further GO enrichment analysis. Compared with the NY-17, more DSGs in the drought-tolerant flax variety Z141 that responded to both DS and RD treatments were enriched in abiotic stress-related pathways (Fig. 2e, f, Additional file 6). These results indicated that the frequency of drought stress-responsive genes regulated by specific AS was higher in Z141 than in NY-17, which may help improve the drought stress tolerance of Z141.

Although studies have shown that AS can improve plant drought resistance by altering transcriptome plasticity [66], other studies have found that DEGs and DSGs minimally overlap in plants under abiotic stress, suggesting that AS and transcriptional regulation are two parallel processes when plants respond to abiotic stresses [71, 72]. This finding is consistent with our observation that only approximately 16–24% of DSGs were regulated by transcription in Z141 and NY-17 under the DS treatment (Fig. 3a). Further GO enrichment analysis also showed that DEG-specific genes in Z41 and NY-17 under the DS treatment were significantly enriched in abiotic stress-related GO terms under the DS treatment, while overlapping DEGs&DSGs and DSGs-specific genes were rarely found to be enriched in related GO terms (Fig. 4, Additional file 11). This finding indicated that the responses of Z141 and NY-17 to drought stress were mainly regulated by transcription. However, most plant drought tolerance studies have been conducted by considering stress as a single event that occurs once in the life of a plant. Therefore, it remains unknown whether repeated drought stress induces different modes of AS regulation in plants. A recent study found that the coordination of transcriptional regulation and AS regulation contributes to resistance to HS and HD in wheat [17]. In this study, we found that over 40% and 32% of DSG gene expression patterns were changed in Z141 and NY-17 under the RD treatment, respectively (Fig. 3a). The results of GO enrichment analysis also showed that many overlapping DSGs and DEGs in Z141 and NY-17 under the RD treatment were significantly enriched in abiotic stress response GO terms (Fig. 3c and Additional file 8). Although the underlying mechanisms of the coordinated regulation of AS and transcription in response to abiotic stresses in plants are unclear, our results strongly suggest that transcriptional regulation may play a major role in linseed response to the DS treatment, which coordinates with AS regulation to contribute to the linseed response to the RD treatment.

Considering the limited number of linseed tissues collected in the experiment and the limitations of RNA-seq sequencing technology, some AS genes may be partially ignored. It is reasonable to assume that the number of AS genes in linseed is underestimated due to technical problems and the inherently dynamic nature of AS in plants. In addition, although the current RNA-Seq technology can identify AS events between two exons, it cannot provide full-length transcript information, including all AS events [73]. Therefore, we also cannot predict the functional changes in genes regulated by AS, which requires further analysis of relevant biological experiments.

DNA methylation is an important epigenetic modification that affects a variety of biological processes and plays a significant role in plant responses to drought stress [74, 75]. However, how the change in methylation of these drought stress-related genes affect the linseed response to drought remains unclear. In this study, we assessed the dynamics of DNA methylation in linseed cultivars, comparing the responses of two cultivars to repetitive drought stress. The overall methylation levels of Z141 and NY-17 seedlings were slightly increased under drought stress (Fig. 5a), which was similar to the findings for other species [26, 76]. Previous studies have shown significant differences in the methylation patterns of specific loci in different cultivars with distinct phenotypes under stress conditions [77]. Our results showed that hypermethylation is more common in drought-tolerant linseed genotypes under drought-stressed and repeated drought-stressed conditions (Fig. 5c). Previous studies have shown that CHH-DMRs are more involved in the regulation of plant development and response to drought stress [78, 79]. Although the number of CHH-DMRs under most treatments in this study was negligible, the number of DMRs at the CHG and CHH sites in Z141 was significantly higher than that in NY-17 under repeated drought stress (Fig. 5d). A similar phenomenon was found in studies of phosphorus-deficient tomatoes [76]. Overall, these results suggest that cultivar-specific changes in DNA methylation and different unknown mechanism(s) may be responsible for the differential stress responses.

The association of DNA methylation changes with DAS has been controversial. In mammalian studies, it has been demonstrated that cotranscriptional AS may generate the AS patterns required for appropriate responses through epigenetic control [80]. The results of studies during the last decade reveal that plant responses to drought stress are transcriptionally regulated at the chromatin level [81–83], suggesting that drought stress-induced AS may be epigenetically controlled. However, we did not observe any correlation between DMRs and DAS genes in this study (Additional file 15), which is similar to some studies in plants and insects [76, 84]. Nevertheless, we still observed an interesting phenomenon in overlapping DMGs and DSGs. We found that the DSGs with only hypermethylation in the gene body region were significantly enriched in abiotic stress response GO terms in drought-tolerant linseed varieties under the RD treatment, while the opposite was true for drought-sensitive linseed varieties. Similar results were also found in animal and human cell lines, suggesting that gene body methylation plays an important role in AS regulation in some specific genes [85]. In recent years, some studies have reported the association between plant DNA methylation changes and DAS to a certain degree and demonstrated the complexity of the relationship between DNA methylation and DAS [86, 87]. The mechanism by which DNA methylation regulates AS remains largely unknown. AS is regulated by various cis-acting regulatory elements and RNA-binding proteins [88]. Most pre-mRNA splicing is usually co-transcriptional, suggesting that epigenetic mechanisms are involved in splicing regulation [89]. Currently, two mechanisms have been proposed to explain how DNA methylation information is transmitted to splicing regulation: ccctc-binding factor (CTCF) and methyl-cg-binding protein 2 (MeCP2) can regulate the elongation rate of Pol II, and heterochromatin protein 1 (HP1) can recruit splicing factors to alternative exons of transcription [85]. Although further research is required to determine the extent to which gene body methylation impacts AS, the results of this study suggest the possibility of a connection between linseed DNA methylation changes and AS in some specific genes under drought stress.

## Conclusions

In summary, our study is the first comprehensive genome-wide analysis of the relationship between linseed methylation changes and AS under drought and repeated drought stress, which may help us understand the drought stress response mechanism in linseed under drought and repeated drought stress from the perspectives of epigenetics and AS. In particular, the different interaction patterns of DEGs and DSGs under drought stress and repeated drought stress may be of interest in the future. In addition, the methylation and AS regulation differences between drought-tolerant and drought-sensitive linseed varieties may be worthy of attention in the future. The results of this study will provide a comprehensive understanding of AS and epigenetics in plant responses to drought stress and repeated drought stress.

## Accession numbers

The raw transcriptome data have been uploaded to the National Center for Biotechnology Information Short Read Archives (https://www.ncbi.nlm.nih.gov/sra/PRJNA598287). MethylC-seq data have been uploaded to the National Center for Biotechnology Information (https://www.ncbi.nlm.nih.gov/geo/query/acc.cgi?acc=GSE213719).

## Availability of and materials

All relevant data can be found within the manuscript and its supporting materials.

## Supplementary Information

**Additional file 1: Table S1.** Statistical summary of read mapping.

**Additional file 2: Table S2.** All AS events identified in Z141 and NY-17 under drought stress.

**Additional file 3: Table S3.** All DSGs identified in Z141 and NY-17 under drought stress.

**Additional file 4: Table S4.** The GO terms of DSGs in Z141 and NY-17.

**Additional file 5: Table S5.** Analysis of differentially expressed genes in linseed seedlings under drought stress.

**Additional file 6: Table S6.** GO analysis of nonoverlapping genes.

**Additional file 7: Figure S1.** GO terms for DSG in different clusters.

**Additional file 8: Figure S2.** Expression and AS analysis of *SR* genes in response to the DS, RW and RD treatments.

**Additional file 9: Figure S3.** REVIGO analysis of overlapping DSGs and DEGs.

**Additional file 10: Figure S4.** Distribution of IEP changes among upregulated, downregulated and non-DEGs under the DS and RD treatments.

**Additional file 11: Figure S5.** Expression and AS analysis of the *SNRK*, *AGB*, and *AMP* genes in Z141 and NY-17.

**Additional file 12: Figure S6.** Functional enrichment analysis of DSG-specific, DEG-specific and overlapping DEGs&DSGs in NY-17.

**Additional file 13: Table S7.** Genome-wide methylation analysis of linseed seedlings under drought stress.

**Additional file 14: Table S8.** Statistics of DMRs of linseed under drought stress.

**Additional file 15: Figure S7.** Correlations between DNA methylation alterations and differential alternative splicing.

## Abbreviations

AS: Alternative splicing
DS: Drought stress
RD: Repeated drought stress
IR: Intron-retention
DMGs: Differentially methylated genes
DSGs: Differentially spliced genes
ALA: α-Linolenic acid
SDG: Secoisolariciresinol diglucoside
MXE: Mutually exclusive exons
ES: Exon skipping
Alt5’SS: Alternative 5’ splicing site
Alt3’SS: Alternative 3’ splicing site
NMD: Nonsense-mediated decay
SMRT: Single-molecule real-time
SGS: Second-generation sequencing
GO: Gene Ontology
ASWC: Absolute soil water content
