# Supplementary material for: The pattern of alternative splicing and DNA methylation alteration and their interaction in linseed (Linum usitatissimum L.) response to repeated drought stresses
Source: Biol Res. 2023 Mar 16;56:12. doi: 10.1186/s40659-023-00424-7 (PMC10018860; doi:10.1186/s40659-023-00424-7)
Supplement: Supplementary file 7 — Additional file 7: Figure S1. GO terms for DSG in different clusters. [file 40659_2023_424_MOESM7_ESM.docx]

(a)


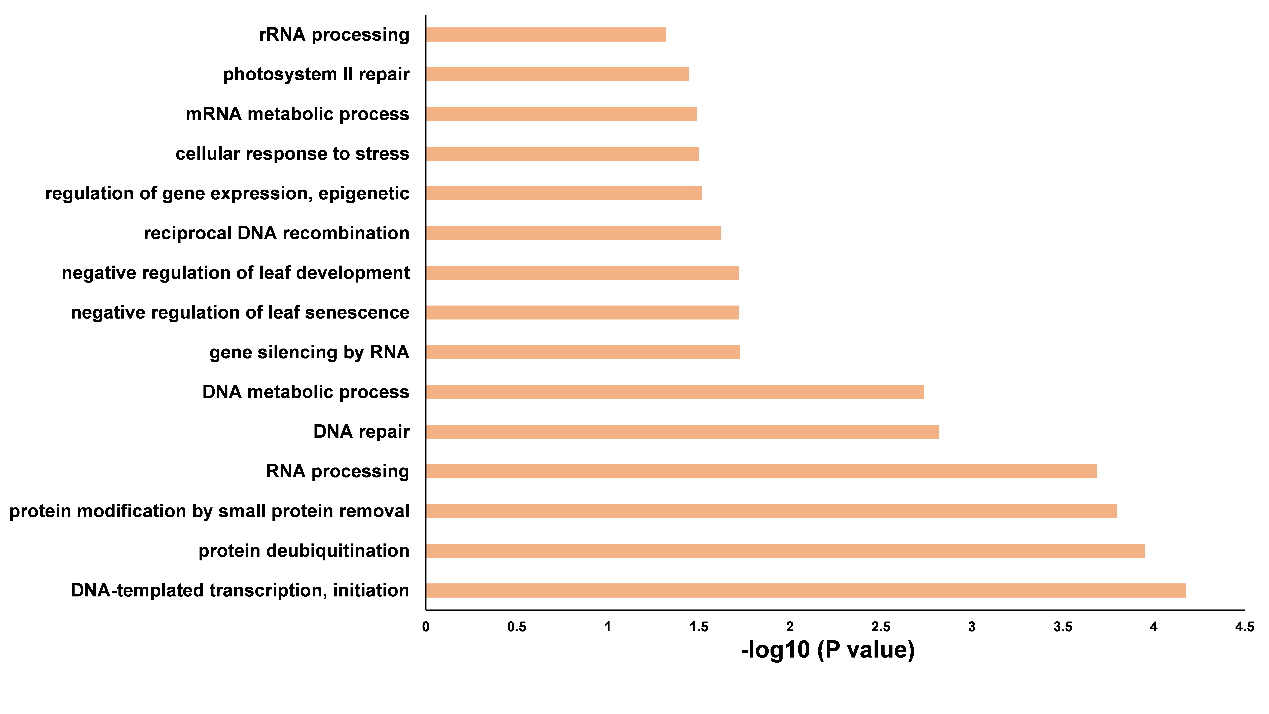


(b)


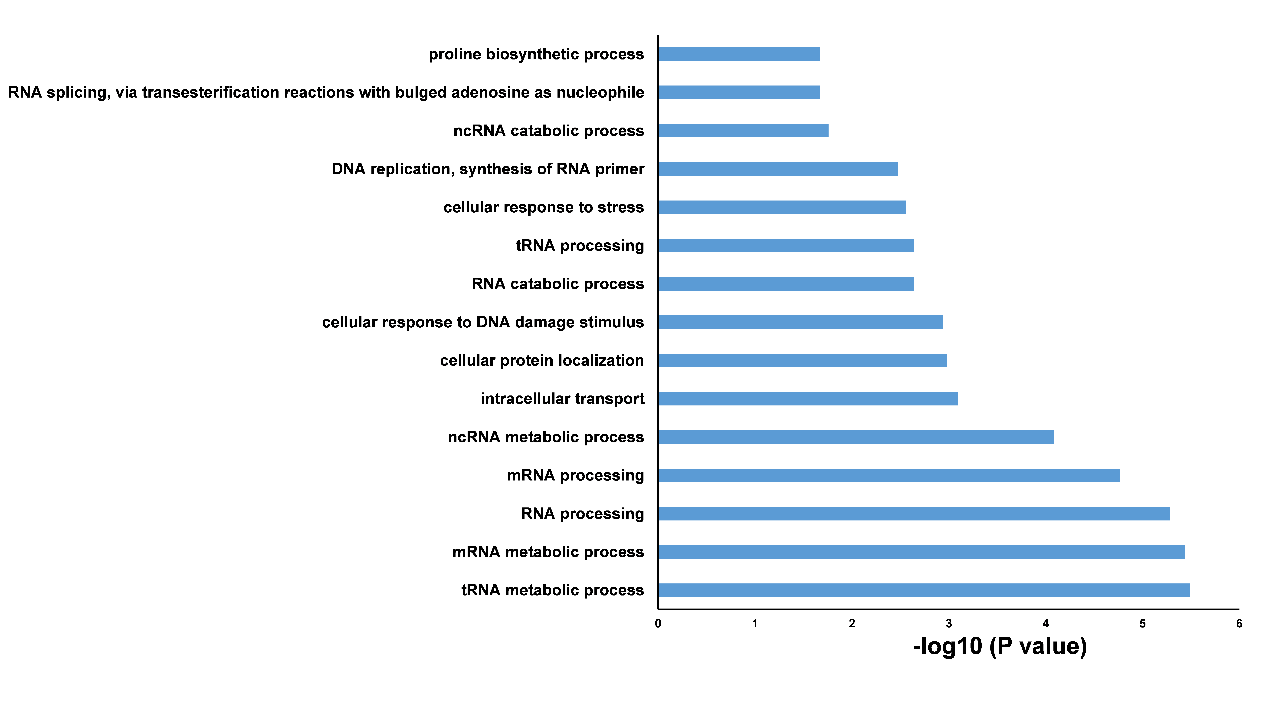


(c)


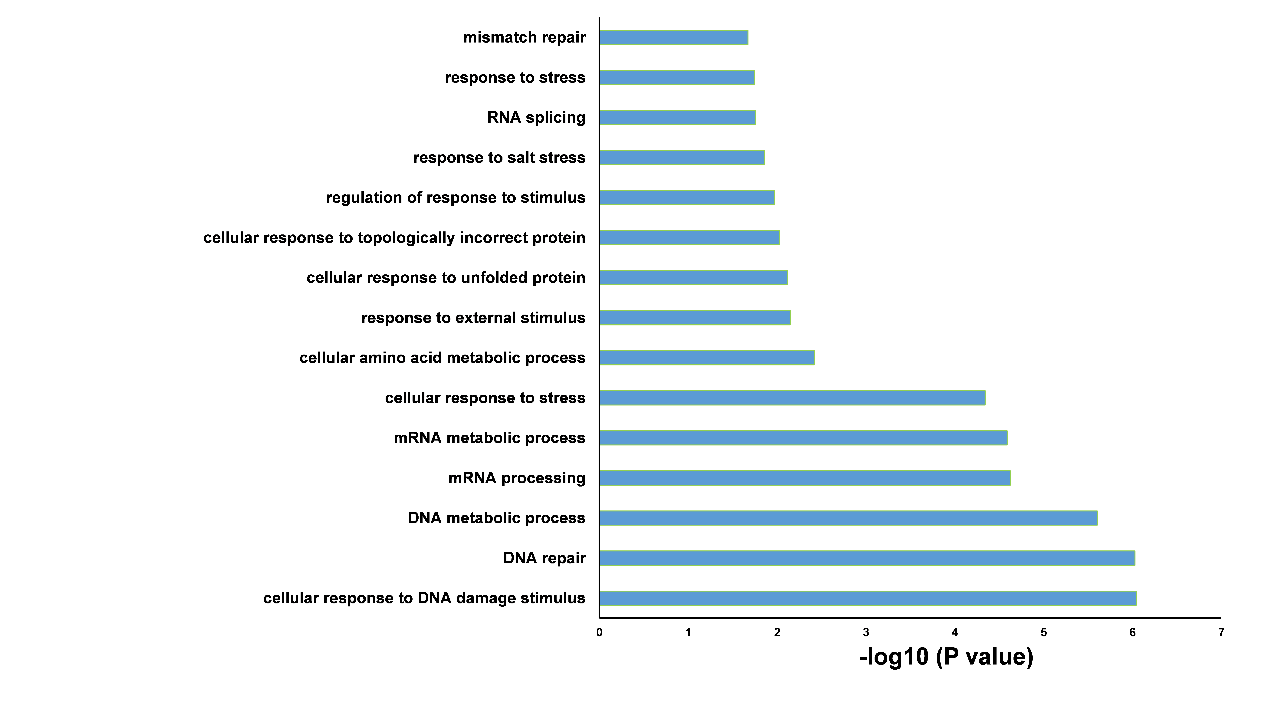


(d)


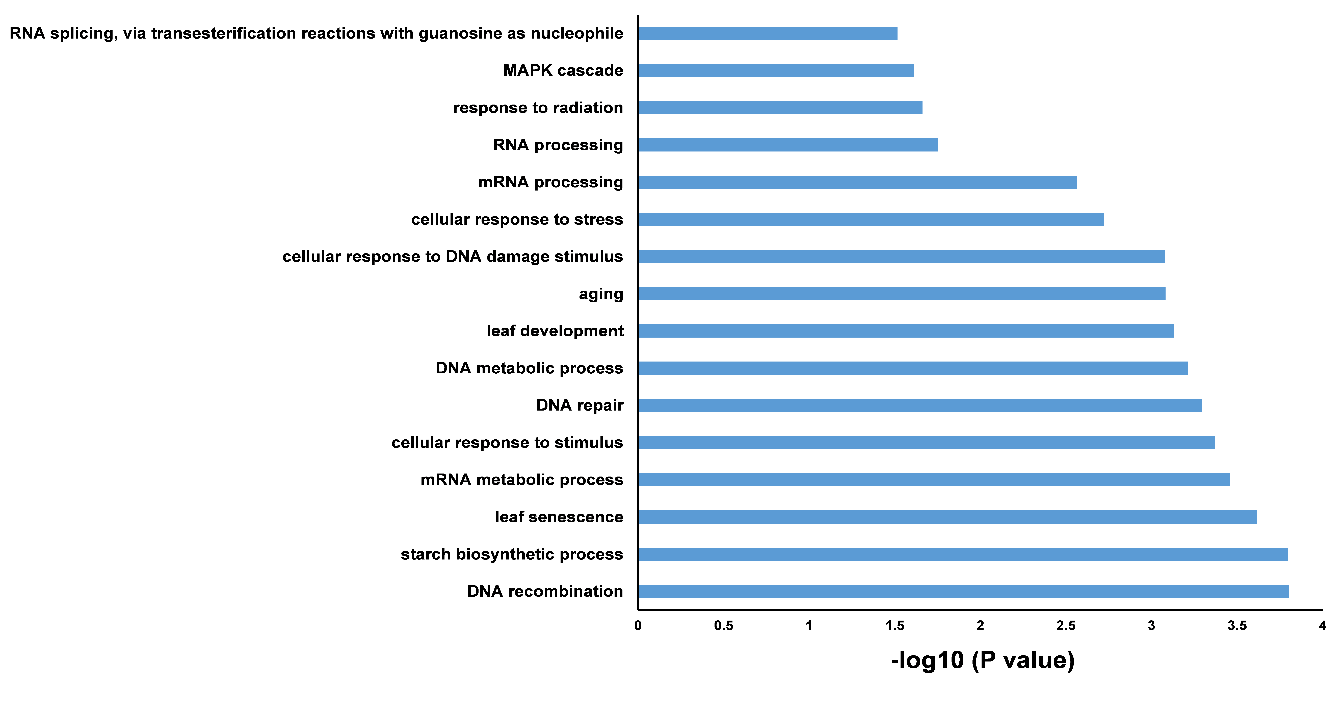


(e)


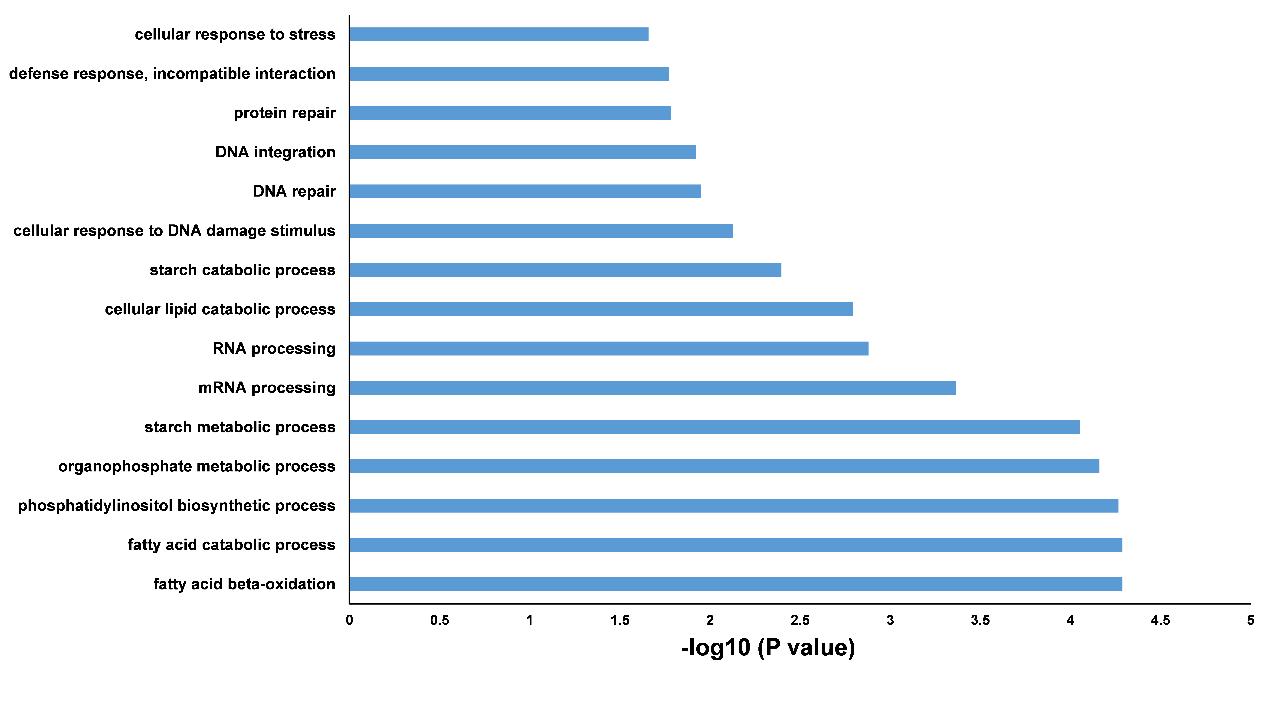


(f)


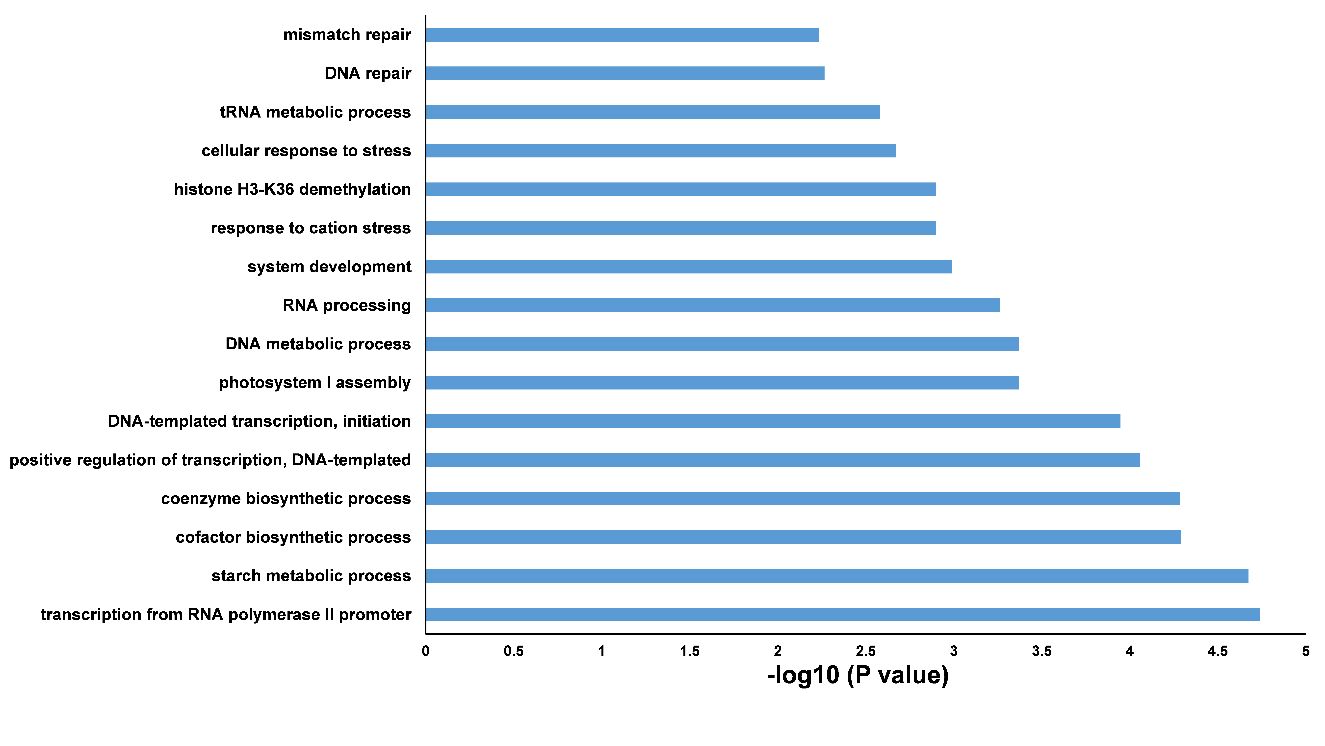


(g)


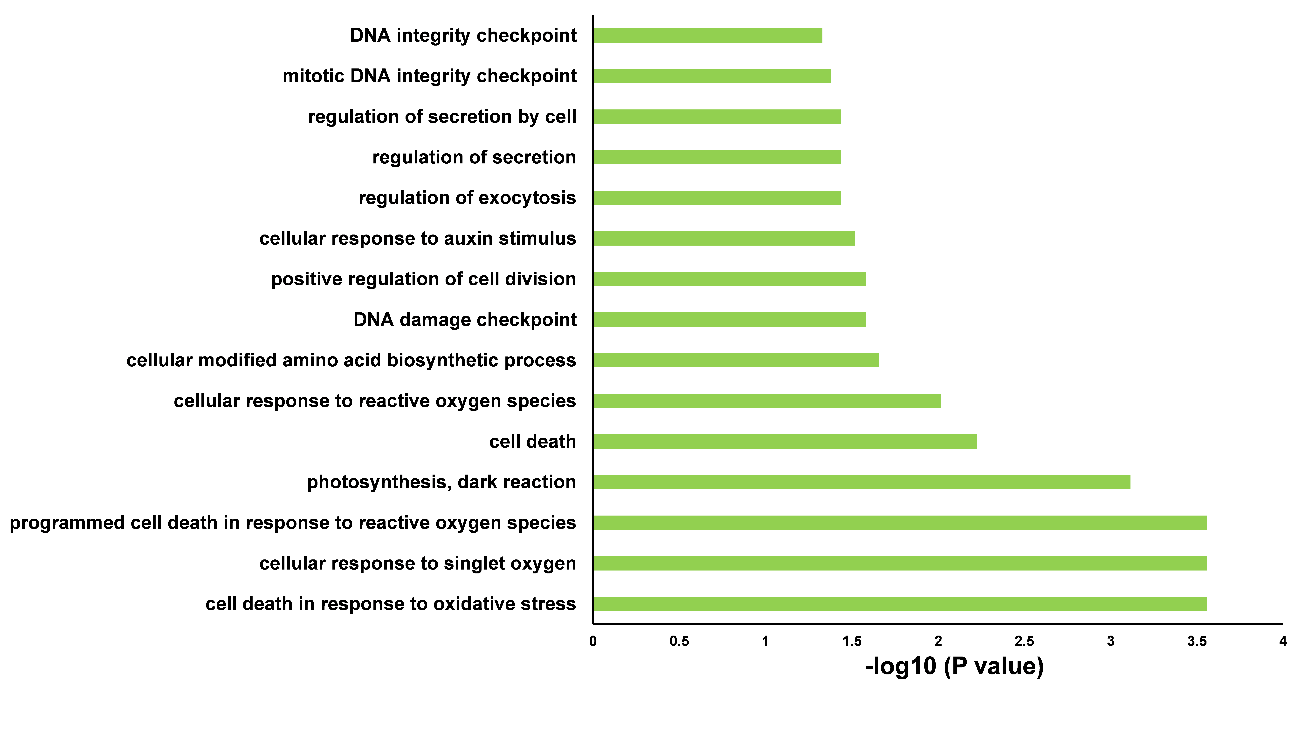


(h)


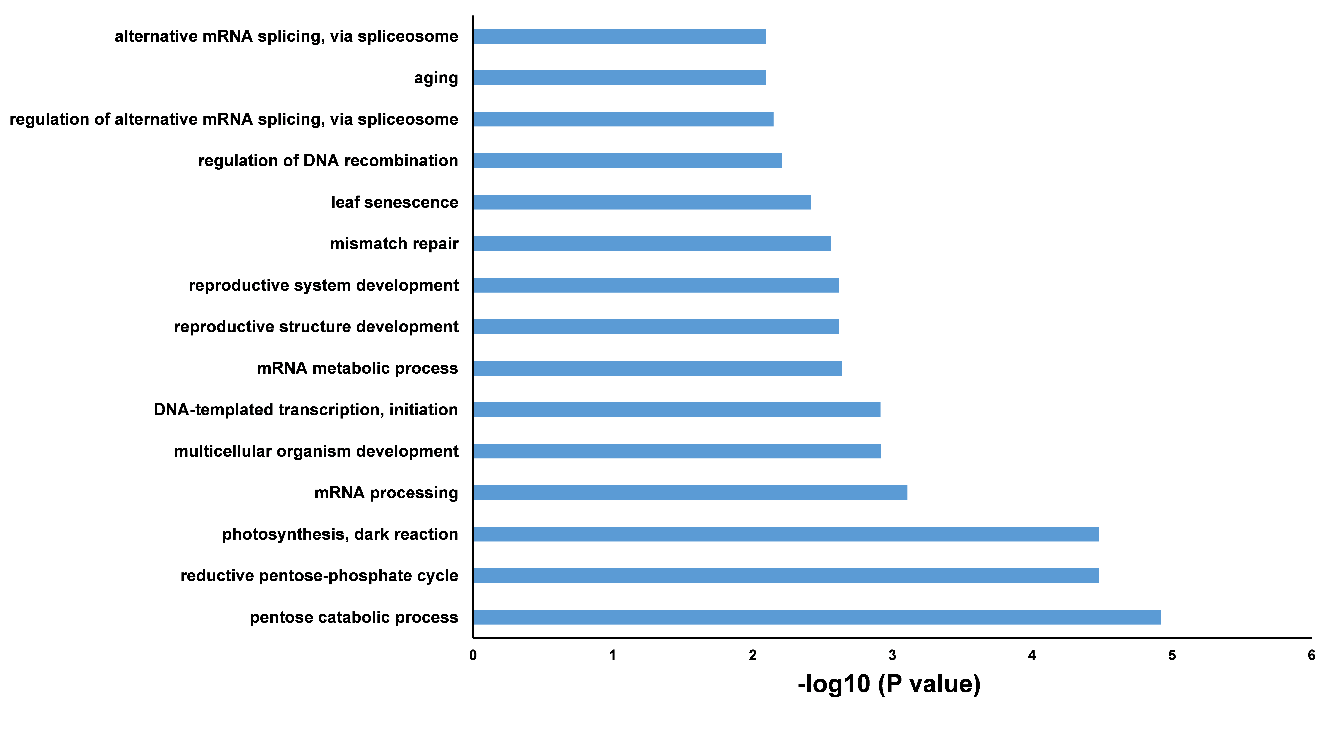


(i)


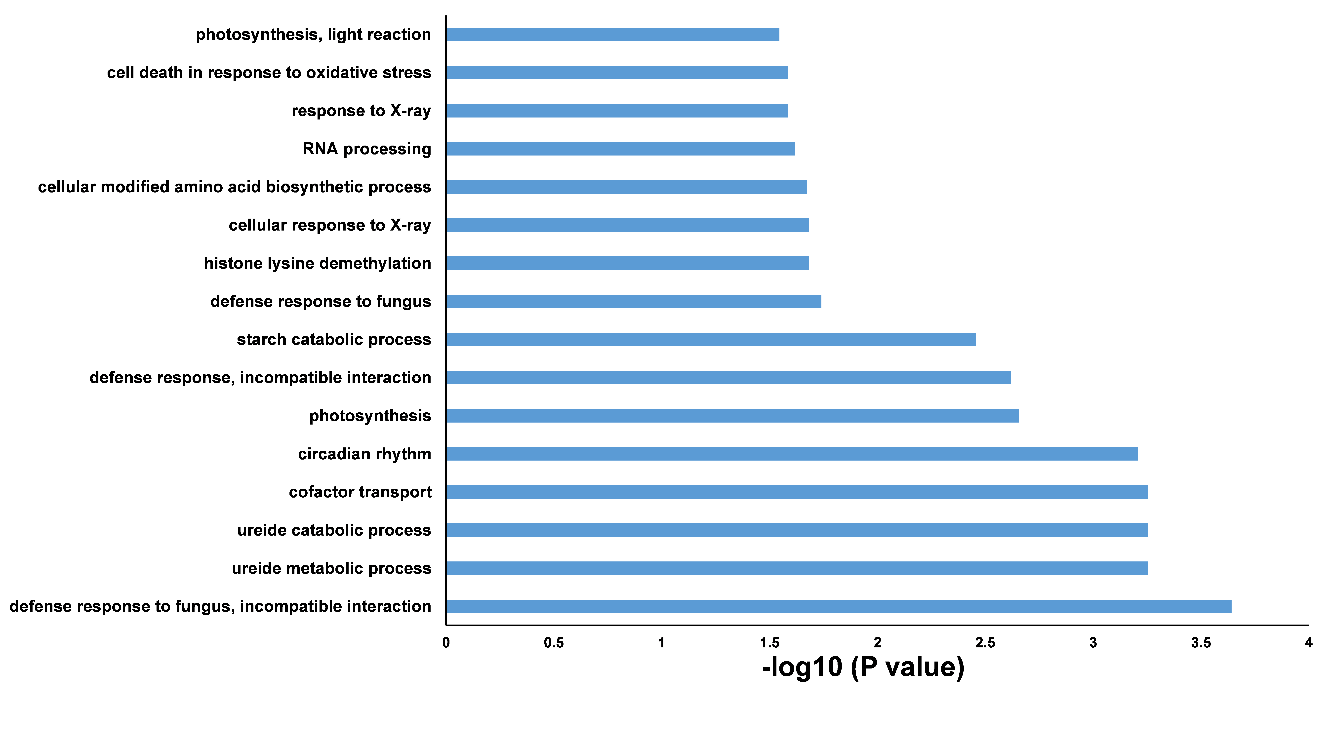


(j)


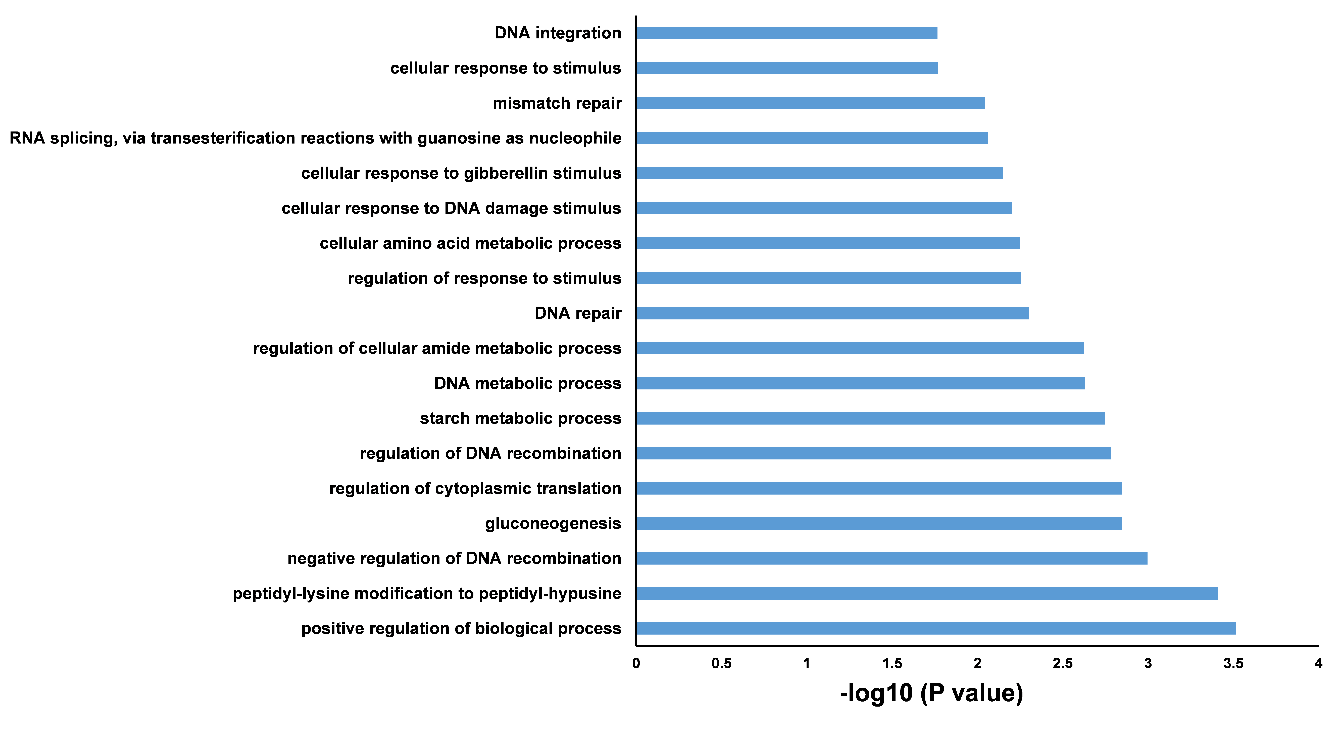


(k)


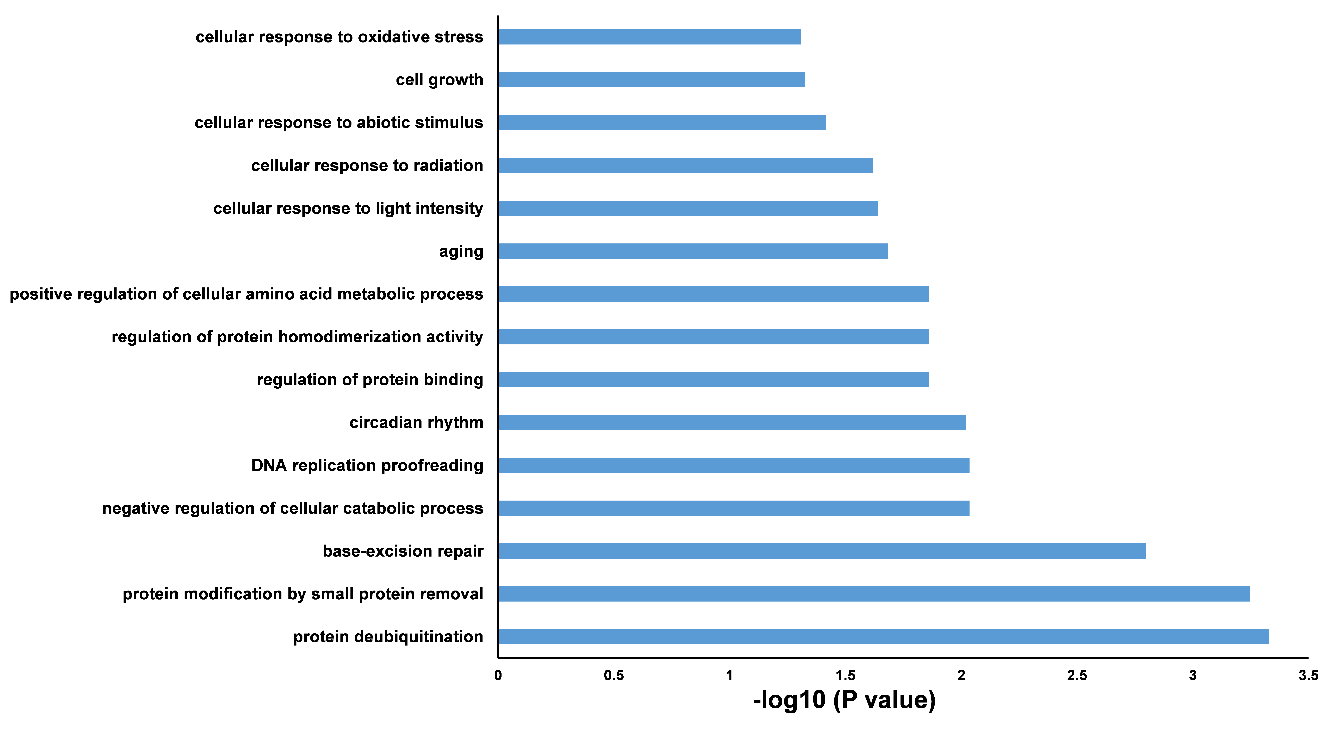


(l)


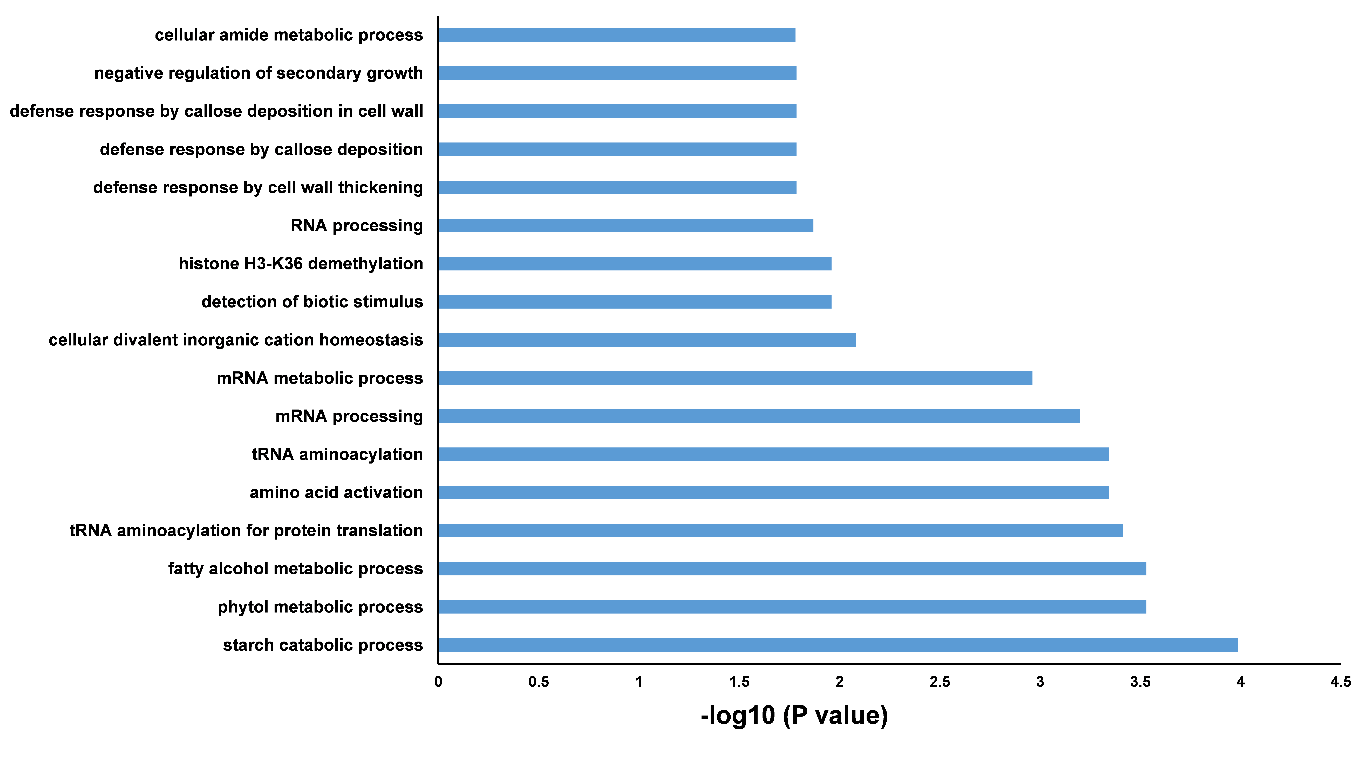


Figure S1. GO terms for DSG in different clusters. (a-f) indicated GO terms for DSG in clusters 1–6 in Z141, respectively. (g-l) indicated GO terms for DSG in clusters 1–6 in NY-17, respectively
