# Supplementary material for: The pattern of alternative splicing and DNA methylation alteration and their interaction in linseed (Linum usitatissimum L.) response to repeated drought stresses
Source: Biol Res. 2023 Mar 16;56:12. doi: 10.1186/s40659-023-00424-7 (PMC10018860; doi:10.1186/s40659-023-00424-7)
Supplement: Supplementary file 8 — Additional file 8: Figure S2. Expression and AS analysis of SR genes in response to the DS, RW and RD treatments. [file 40659_2023_424_MOESM8_ESM.docx]

**(a)**


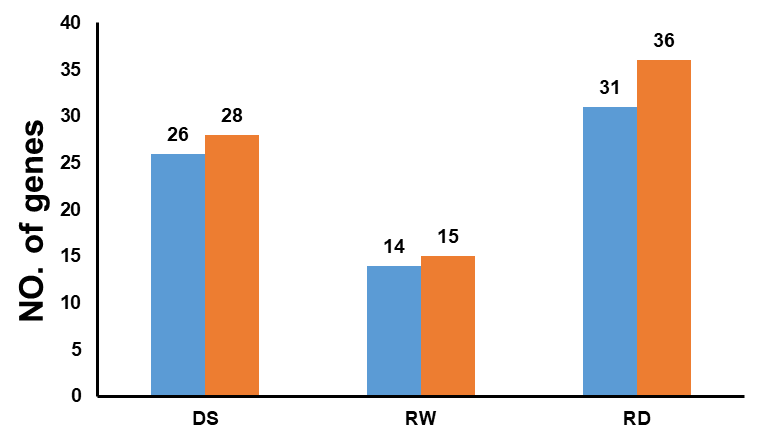


**(b)**


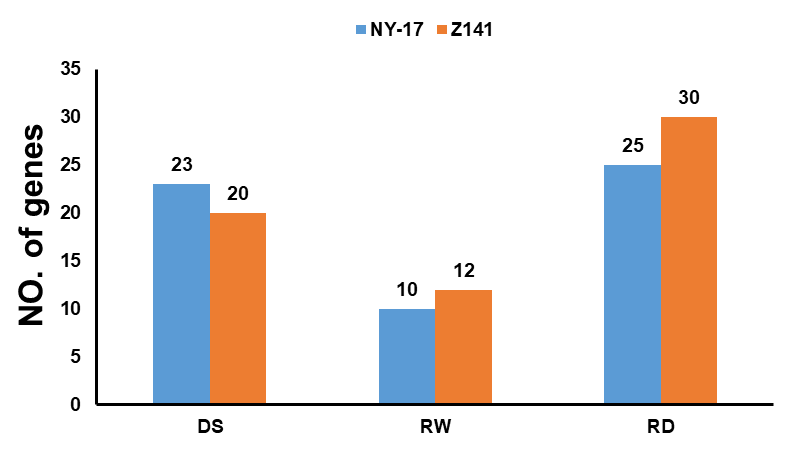


Figure S2. Expression and AS analysis of *SR* genes in response to DS, RW and RD treatments. (a) The number of differentially expressed *SR* genes identified after DS, RW, and RD treatments.

(b) The number of differentially spliced *SR* genes identified after DS, RW, and the RD treatments.
