# Supplementary material for: The pattern of alternative splicing and DNA methylation alteration and their interaction in linseed (Linum usitatissimum L.) response to repeated drought stresses
Source: Biol Res. 2023 Mar 16;56:12. doi: 10.1186/s40659-023-00424-7 (PMC10018860; doi:10.1186/s40659-023-00424-7)
Supplement: Supplementary file 9 — Additional file 9: Figure S3. REVIGO analysis of overlapping DSGs and DEGs. [file 40659_2023_424_MOESM9_ESM.docx]

(a)


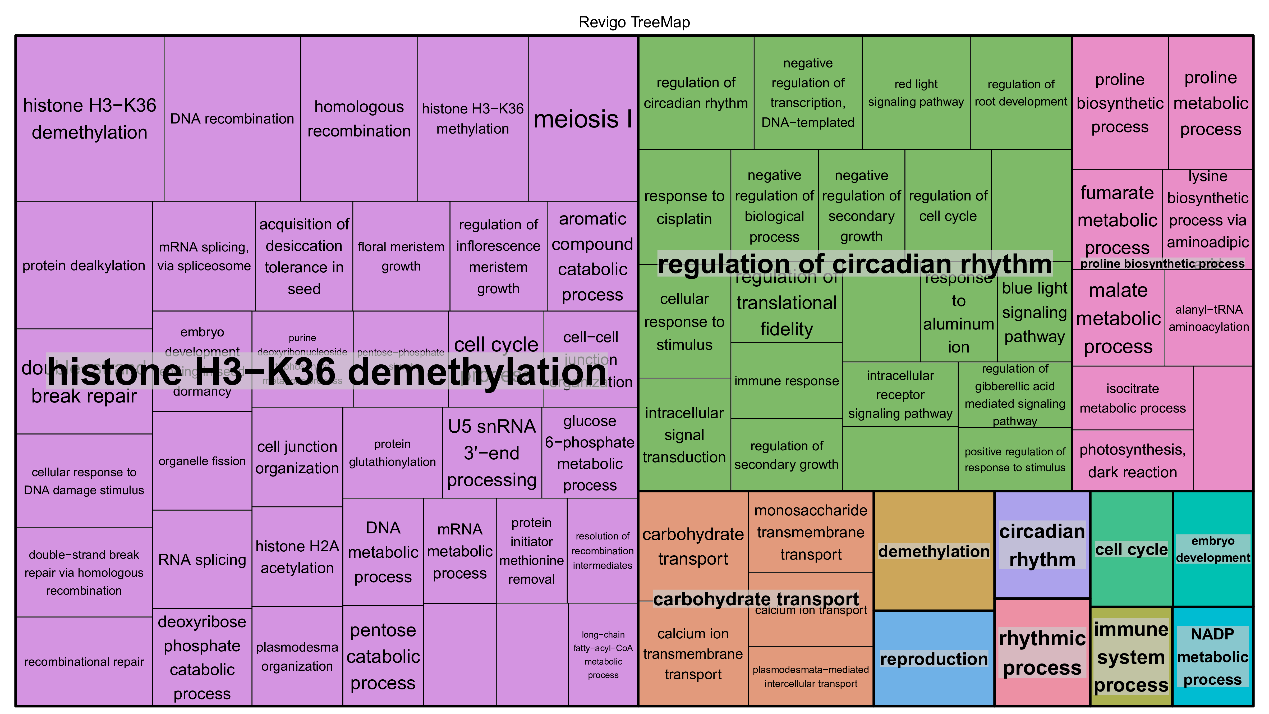


(b)


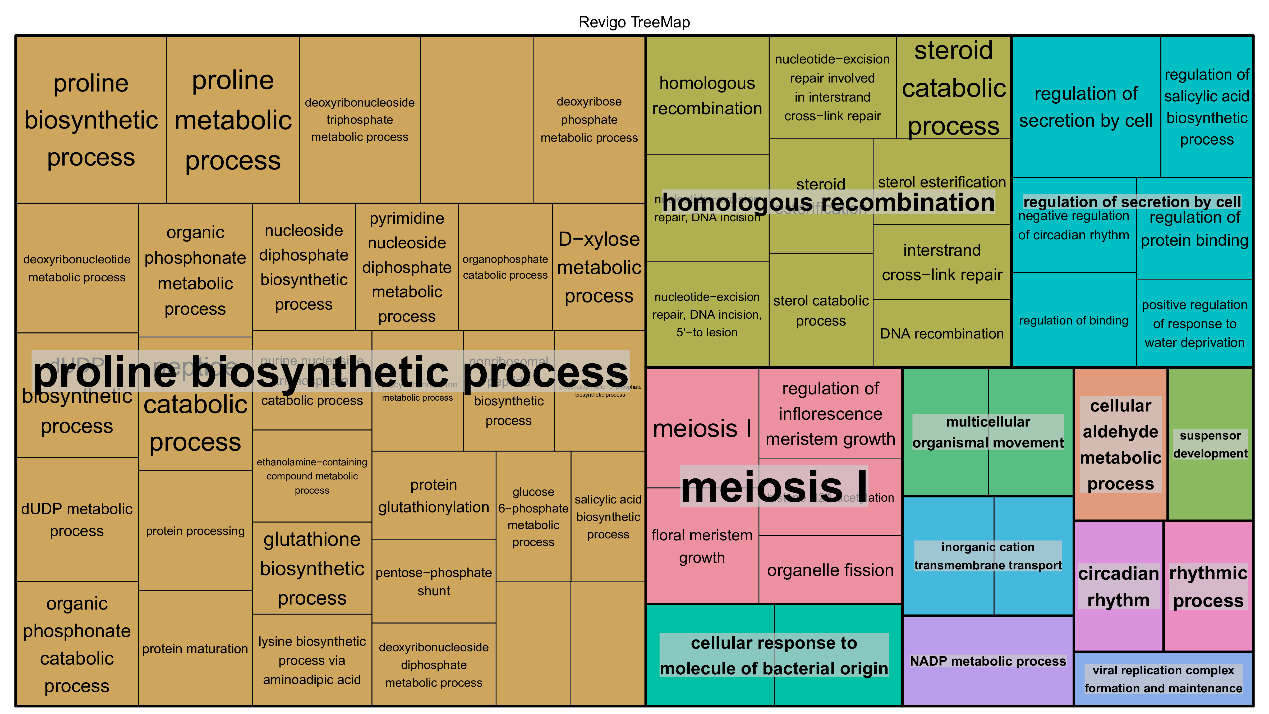


(c)


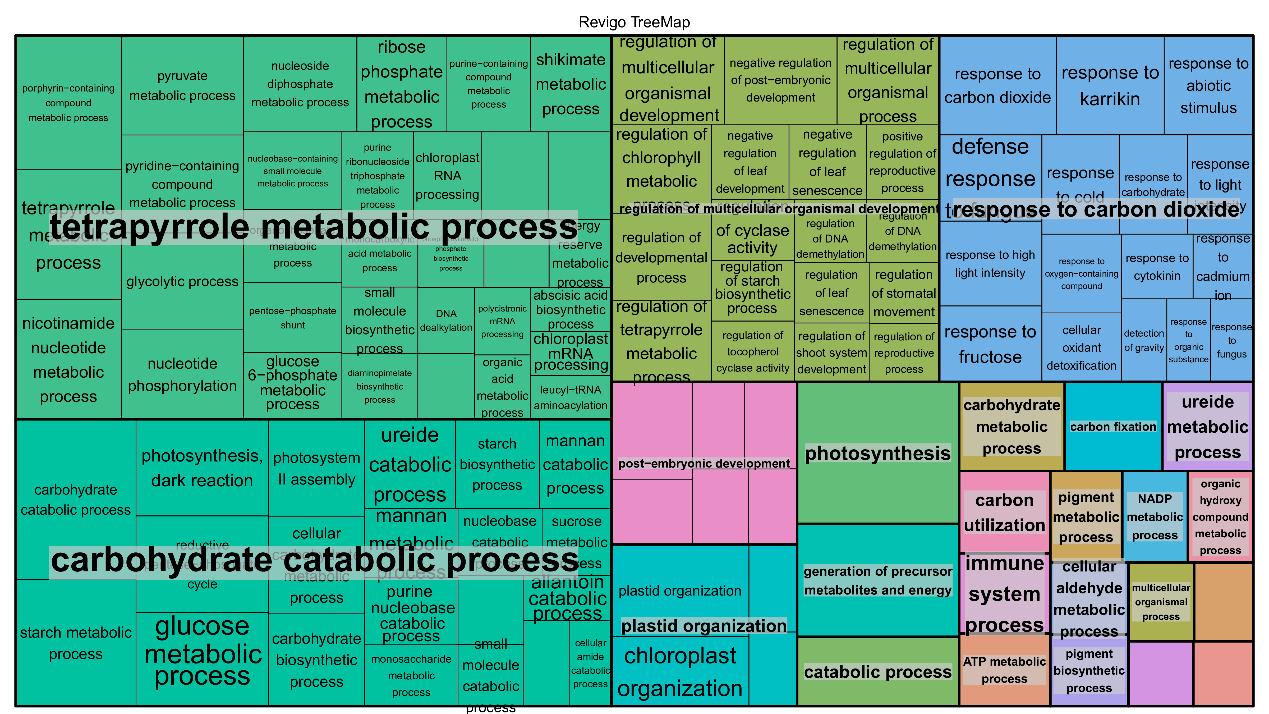


(d)


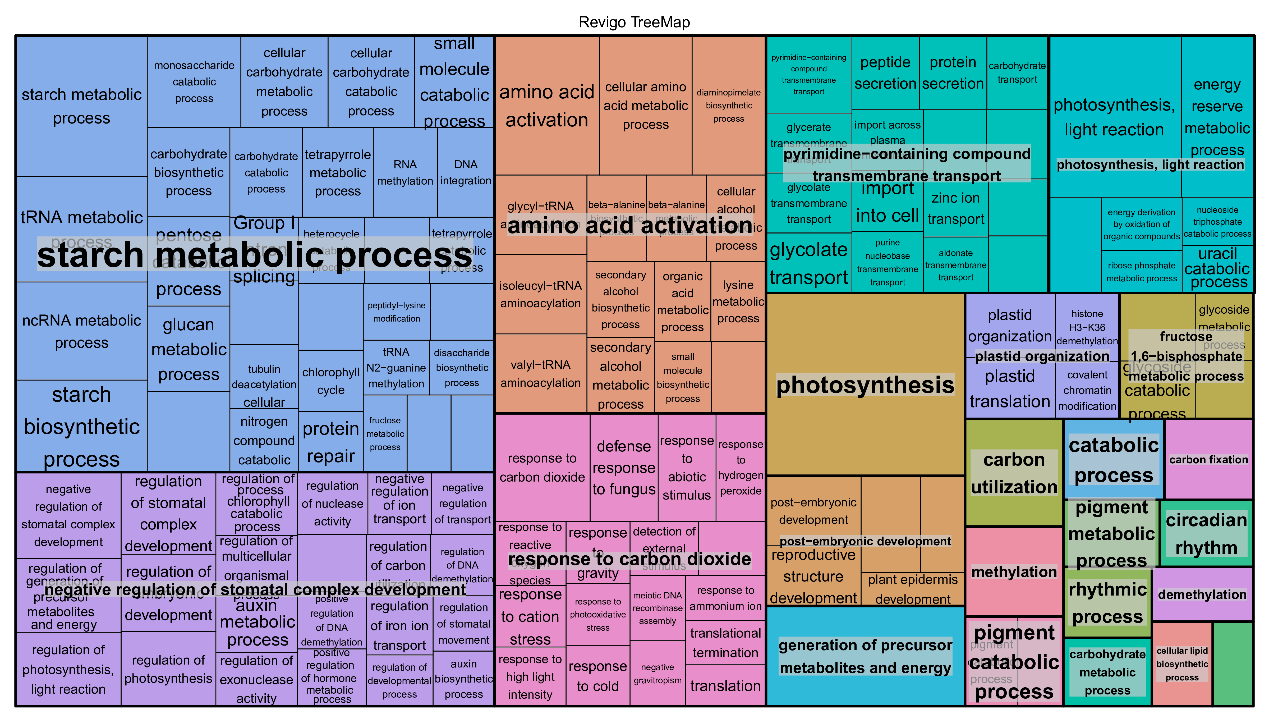


(e)


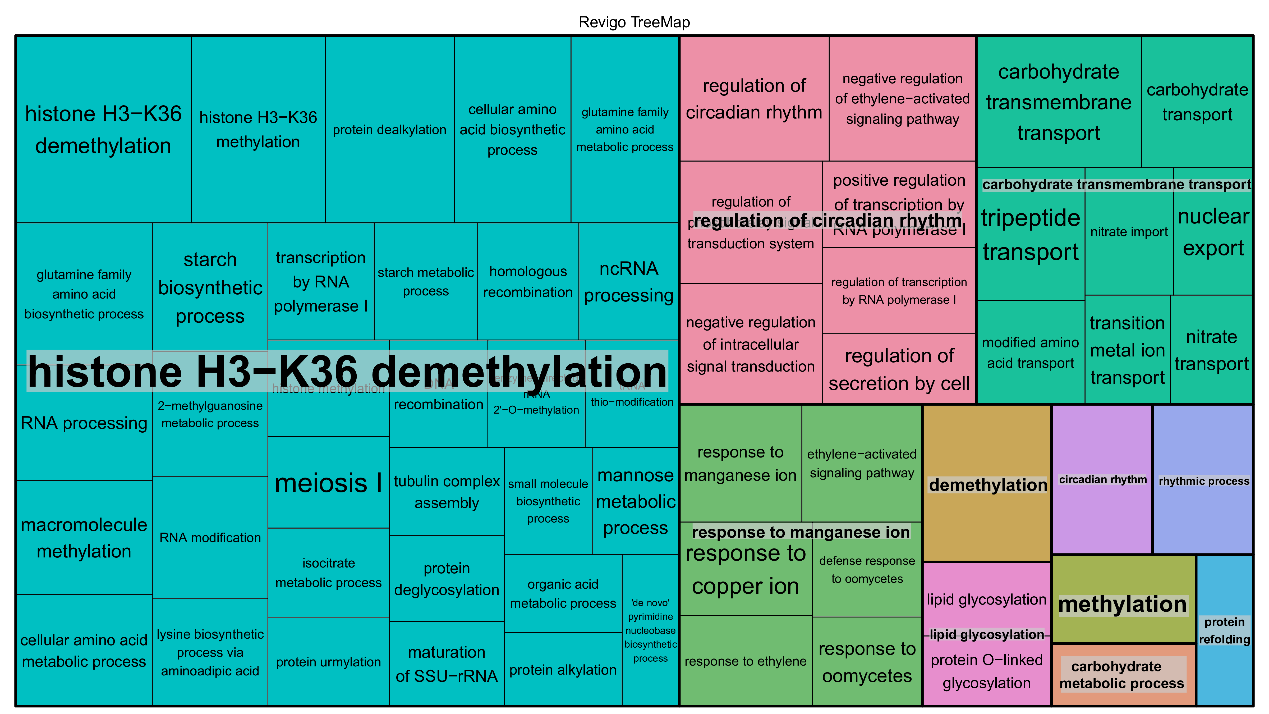


(f)


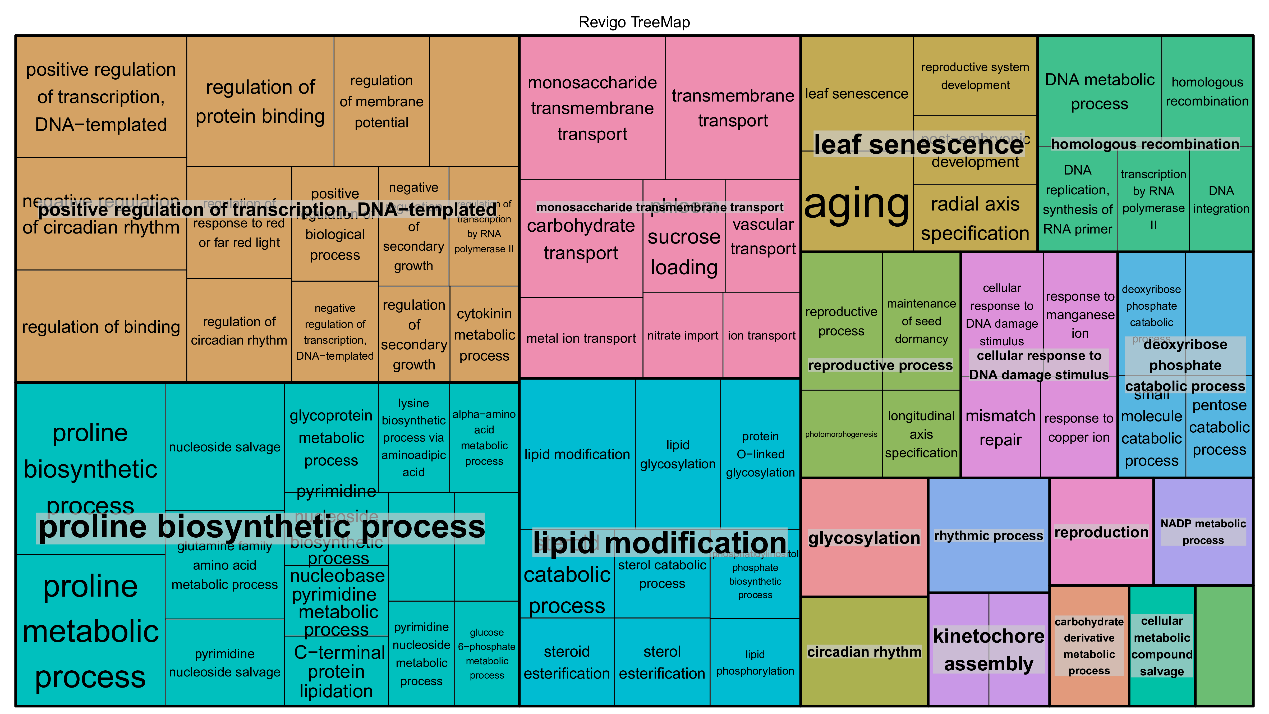


(g)


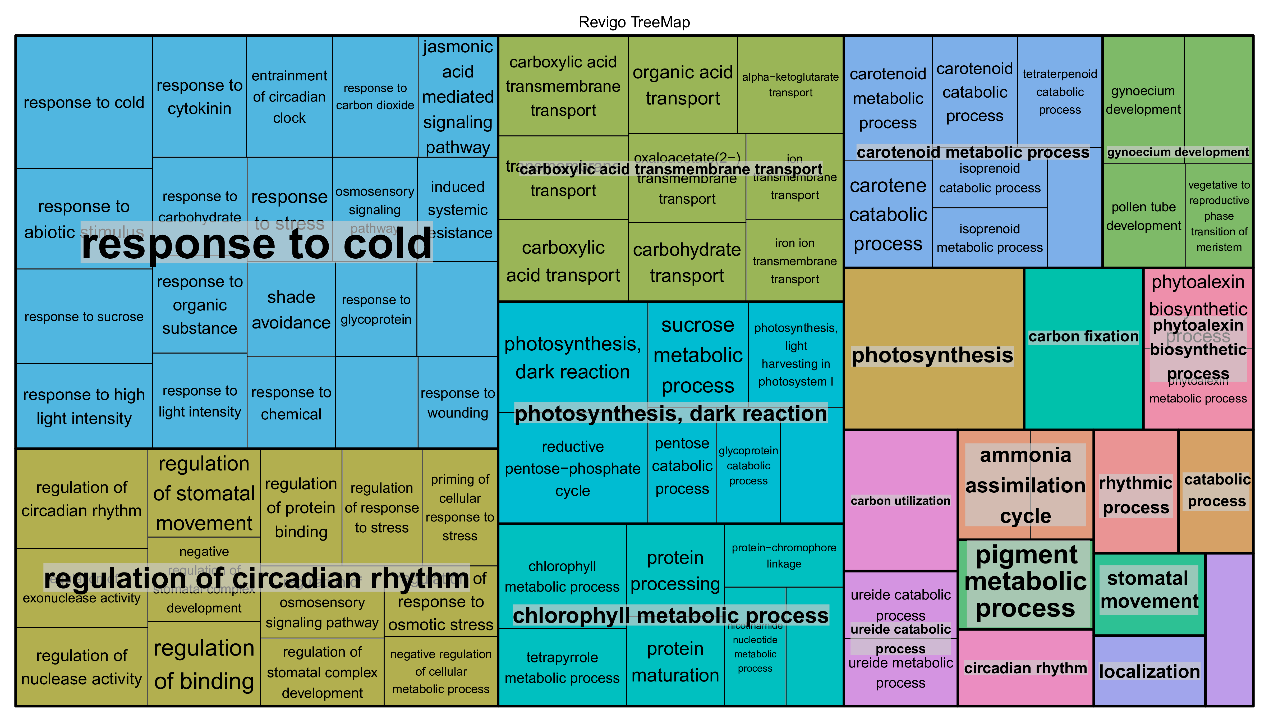


(h)


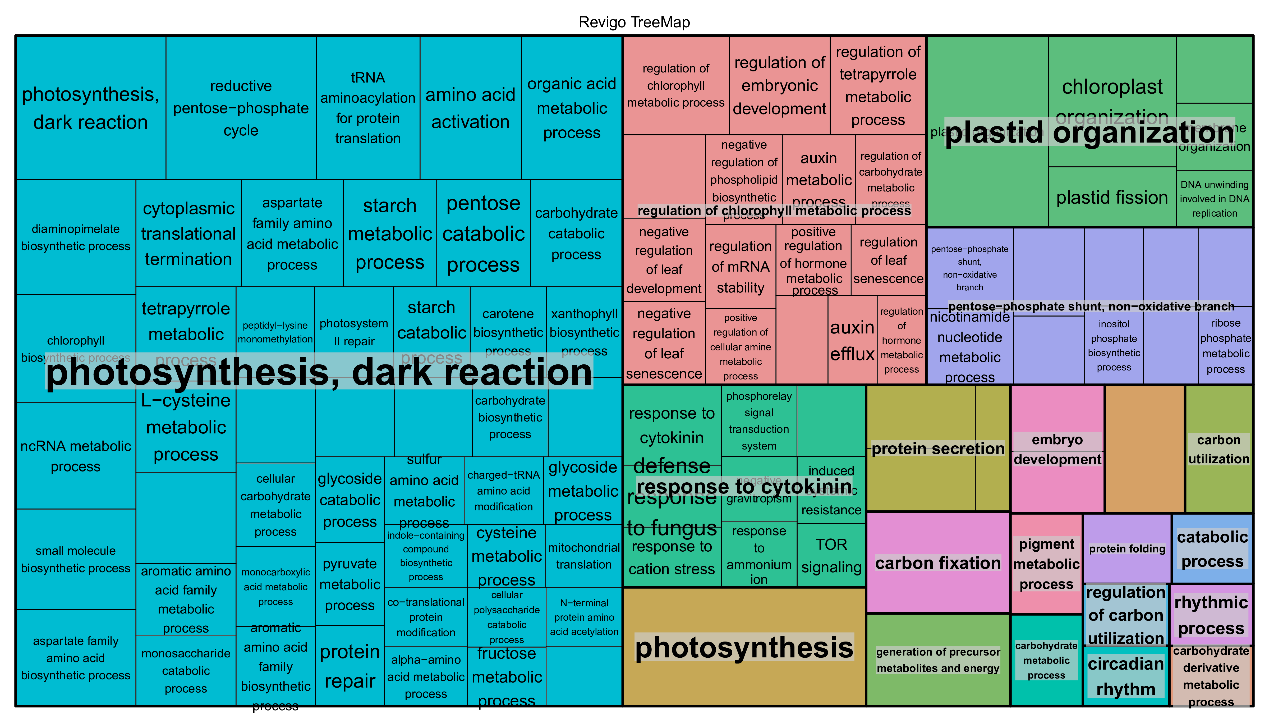


**Figure S3. REVIGO analysis of overlapping DSGs and DEGs. (a-b) The tree diagrams indicate the REVIGO analysis results of upregulated DSGs in Z141 under DS and RD treatments. (c-d) The tree diagrams indicate the REVIGO analysis results of downregulated DSGs in Z141 under DS and RD treatments. (e-f) The tree diagrams indicate the REVIGO analysis results of upregulated DSGs in NY-17 under DS and RD treatments. (g-h) The tree diagrams indicate the REVIGO analysis results of downregulated DSGs in NY-17 under DS and RD treatments.**
