# Supplementary material for: The pattern of alternative splicing and DNA methylation alteration and their interaction in linseed (Linum usitatissimum L.) response to repeated drought stresses
Source: Biol Res. 2023 Mar 16;56:12. doi: 10.1186/s40659-023-00424-7 (PMC10018860; doi:10.1186/s40659-023-00424-7)
Supplement: Supplementary file 10 — Additional file 10: Figure S4. Distribution of IEP changes among upregulated, downregulated and non-DEGs under the DS and RD treatments. [file 40659_2023_424_MOESM10_ESM.docx]

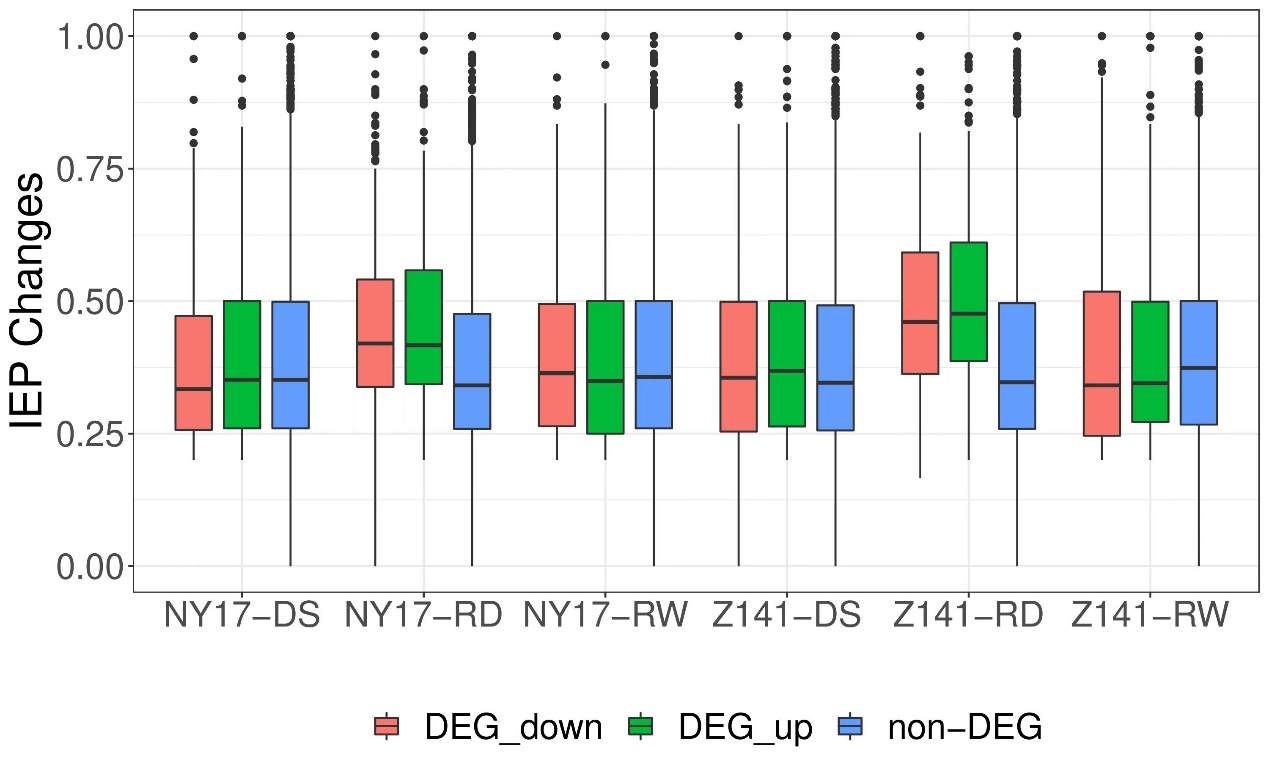


**Figure S4. Distribution of IEP changes among upregulated, downregulated and non-DEGs under the DS and RD treatments.**

**The global IEP change is higher in DEGs than non-DEGs inZ141 and NY-17 under RD treatment.**
