# Supplementary material for: The pattern of alternative splicing and DNA methylation alteration and their interaction in linseed (Linum usitatissimum L.) response to repeated drought stresses
Source: Biol Res. 2023 Mar 16;56:12. doi: 10.1186/s40659-023-00424-7 (PMC10018860; doi:10.1186/s40659-023-00424-7)
Supplement: Supplementary file 11 — Additional file 11: Figure S5. Expression and AS analysis of the SNRK, AGB, and AMP genes in Z141 and NY-17. [file 40659_2023_424_MOESM11_ESM.docx]

**(a)
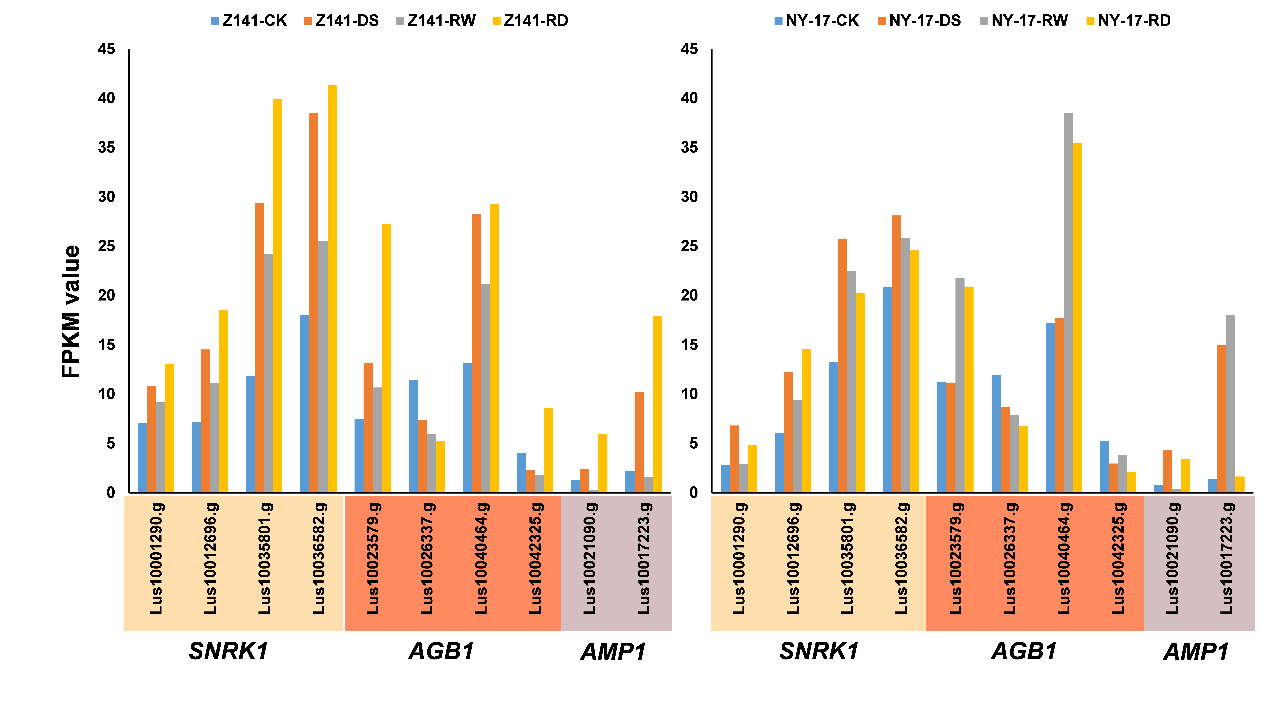
**

**(b)**


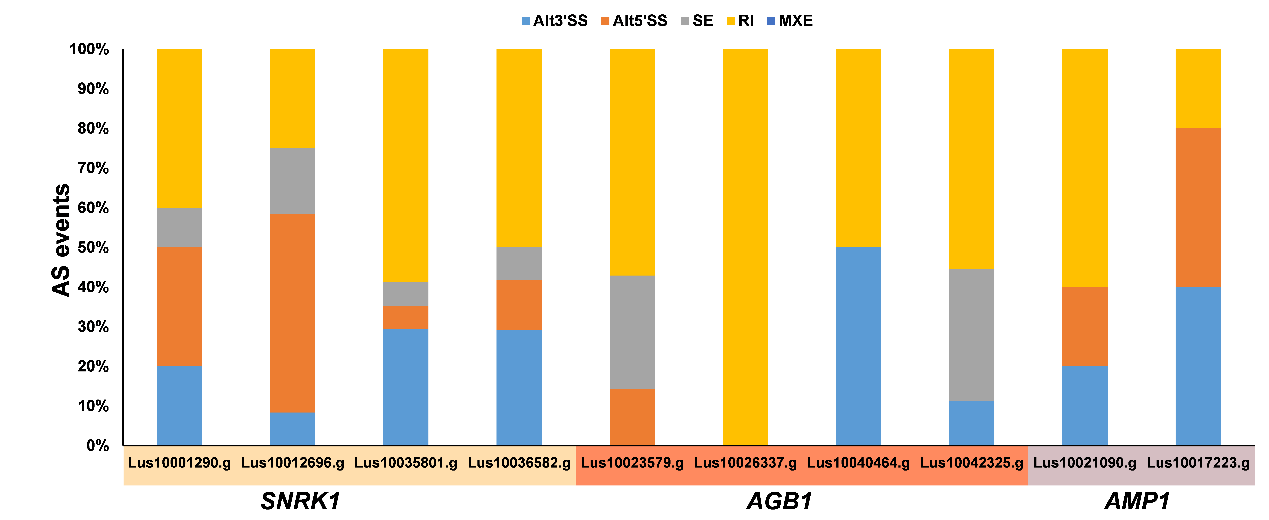


**Figure S5. Expression and AS analysis of the *SNRK1*, *AGB1*, and *AMP1* genes in Z141 and NY-17. (a)The Differential expression level of *SNRK1*, *AGB1*, and *AMP1* genes in Z141 and NY-17 under CK, DS, RW, and RD treatments. (b) The percentage of different splicing event types of *SNRK1*, *AGB1*, and *AMP1* genes in Z141 under RD treatment.**
