# Supplementary material for: The pattern of alternative splicing and DNA methylation alteration and their interaction in linseed (Linum usitatissimum L.) response to repeated drought stresses
Source: Biol Res. 2023 Mar 16;56:12. doi: 10.1186/s40659-023-00424-7 (PMC10018860; doi:10.1186/s40659-023-00424-7)
Supplement: Supplementary file 12 — Additional file 12: Figure S6. Functional enrichment analysis of DSG-specific, DEG-specific and overlapping DEGs&DSGs in NY-17. [file 40659_2023_424_MOESM12_ESM.docx]

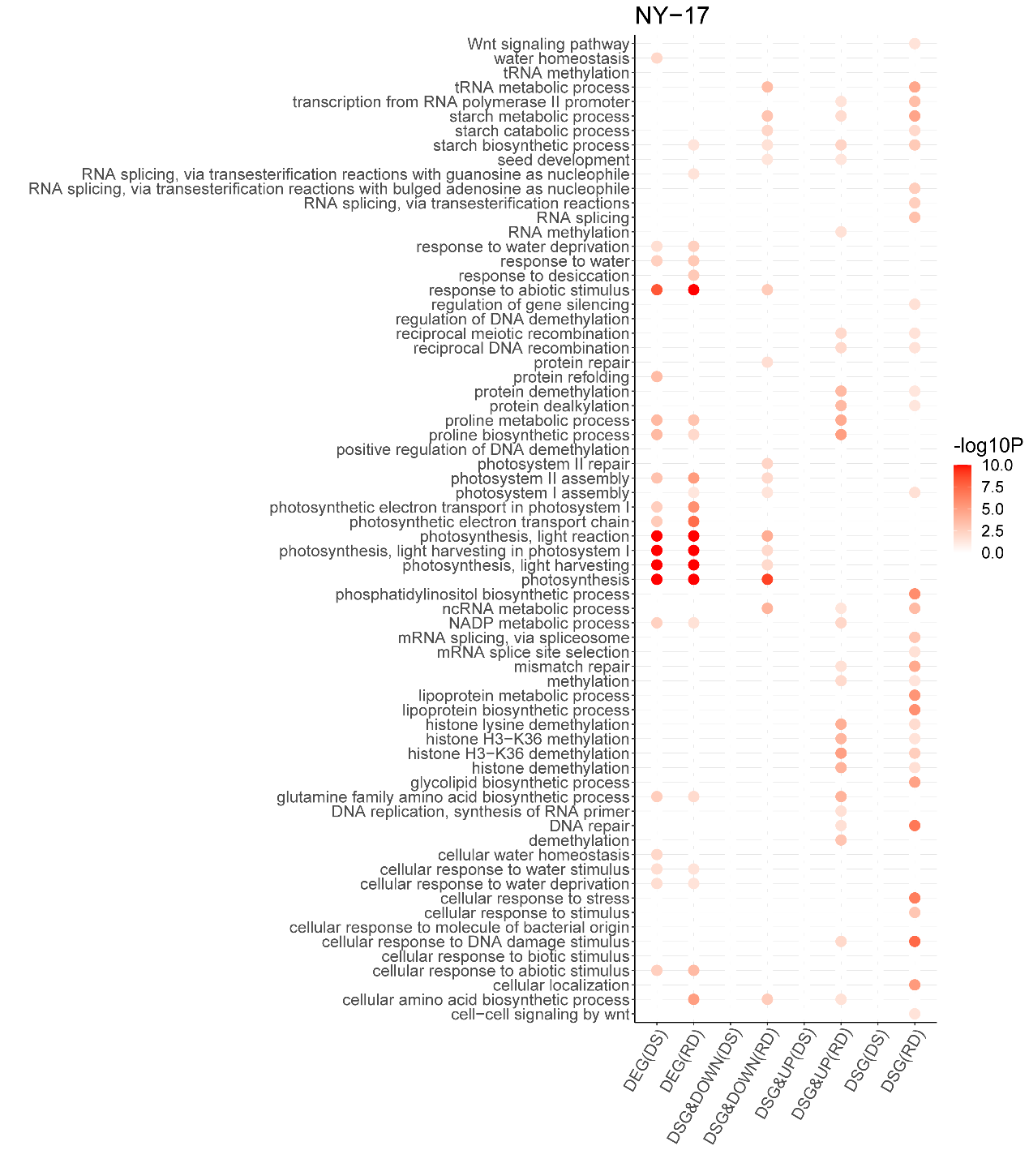


**Figure S6. Functional enrichment analysis of DSG-specific, DEG-specific and overlapping DEGs&DSGs in NY-17. The enriched Gene Ontology (GO) terms of DSGspecific, DEG-specific and DSG&DEG-overlapped genes are shown in bubble diagram. DSG: DSG-specific genes; DSG&Up: genes identified to be both differentially spliced and up-regulated; DSG&Down: genes identified to be both differentially spliced and down-regulated; DEG: DEG-specific genes; DS: drought stress treatment; RD: repeated drought stress treatment.**
