# Supplementary material for: The pattern of alternative splicing and DNA methylation alteration and their interaction in linseed (Linum usitatissimum L.) response to repeated drought stresses
Source: Biol Res. 2023 Mar 16;56:12. doi: 10.1186/s40659-023-00424-7 (PMC10018860; doi:10.1186/s40659-023-00424-7)
Supplement: Supplementary file 15 — Additional file 15: Figure S7. Correlations between DNA methylation alterations and differential alternative splicing. [file 40659_2023_424_MOESM15_ESM.docx]

**(a)**


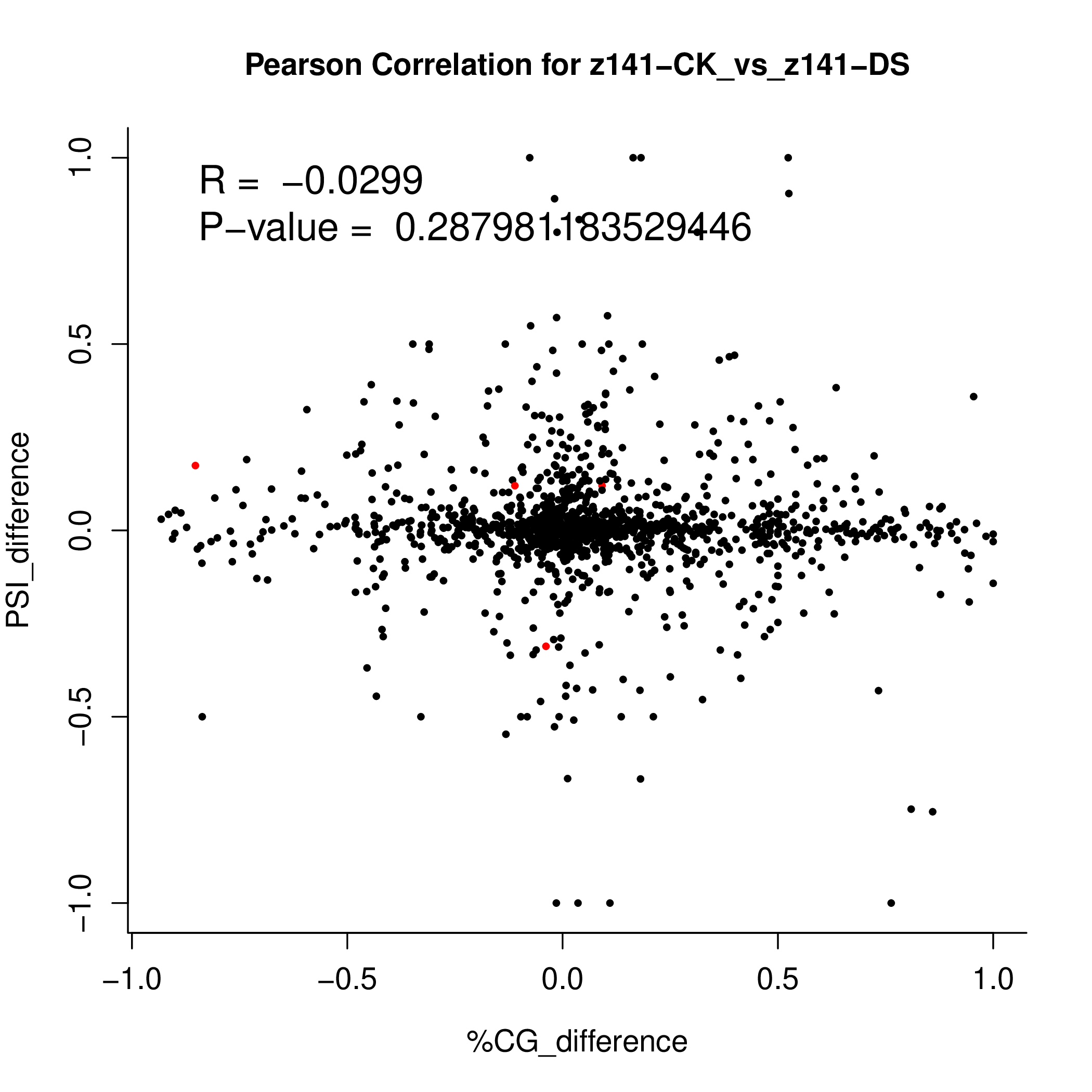


**(b)**


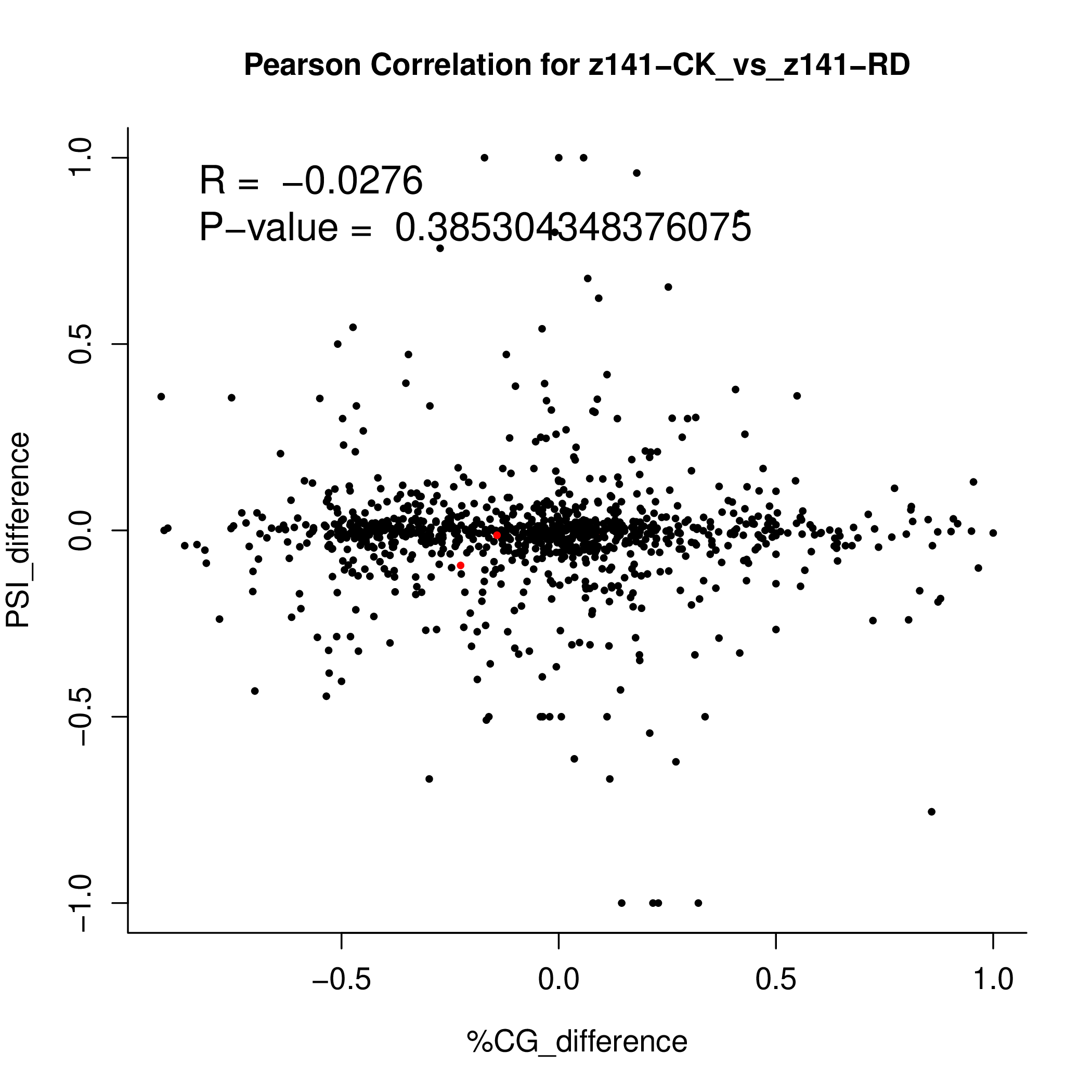


**(c)**


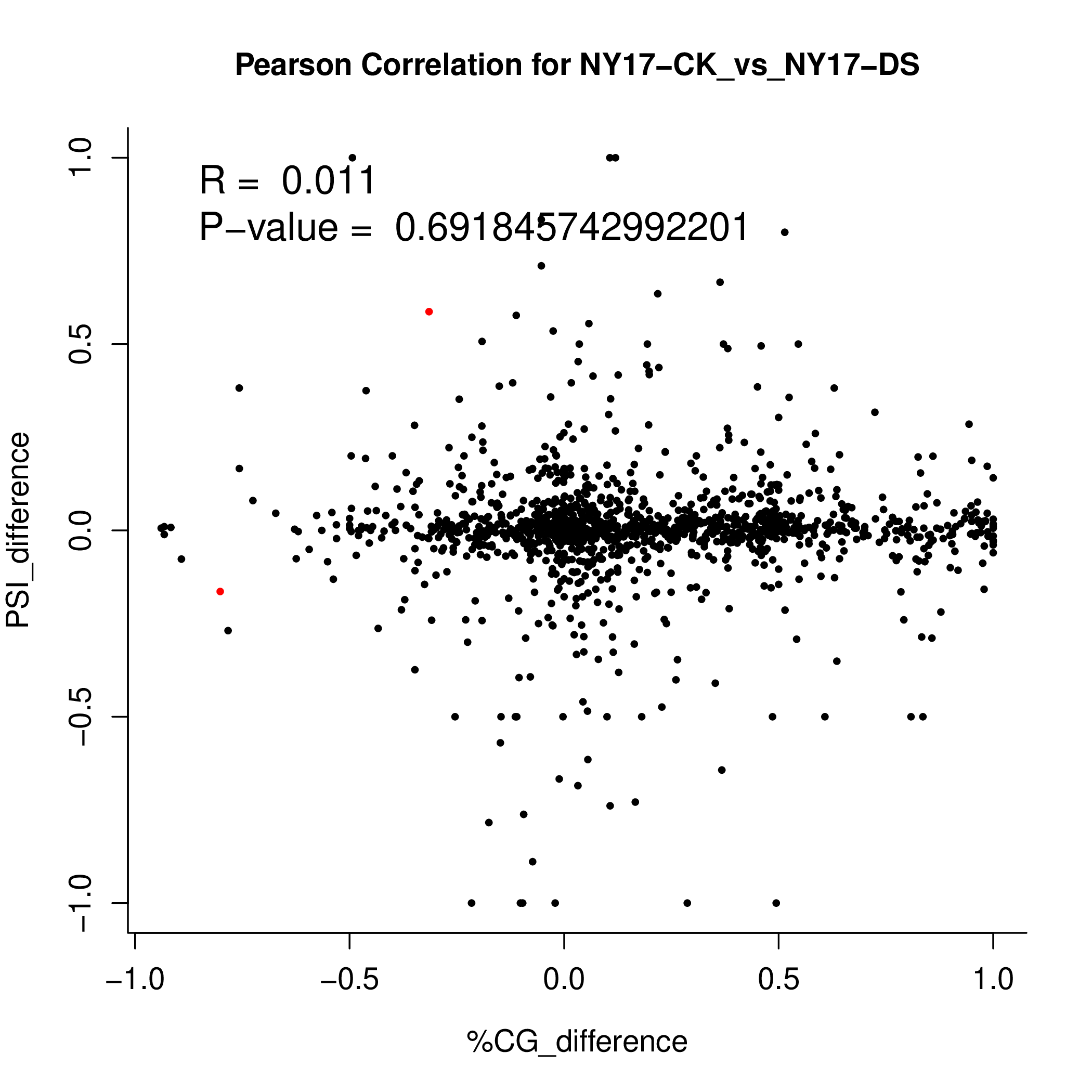


**(d)**


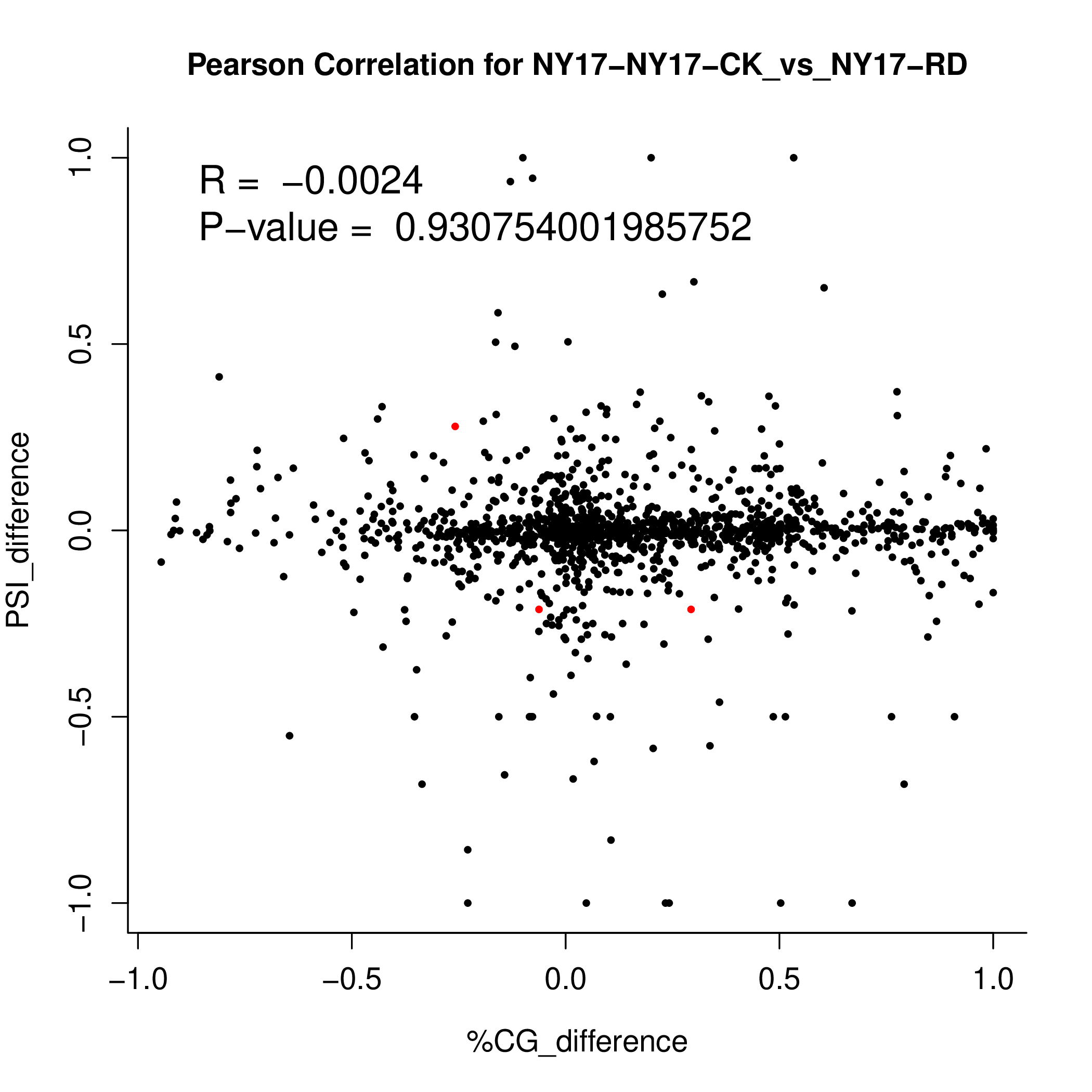


**Figure S7. Correlations between DNA methylation alterations and differential alternative splicing. (a-d) indicated correlations between DNA methylation alterations and differential alternative splicing in Z141 and NY-17 under DS and RD treatments, respectively.**
